# The effects of antioxidants on knee osteoarthritis: A systematic review and meta-analysis

**DOI:** 10.3389/fnut.2022.1026450

**Published:** 2022-12-19

**Authors:** Mohammad Nejadhosseinian, Shirin Djalalinia, Hoda Haerian, Majid Alikhani, Asieh Mansour, Amir-Hossein Mousavian, Heydar Ali Mardani-Fard, Amir Kasaeian, Seyedeh Tahereh Faezi

**Affiliations:** ^1^Joint Reconstruction Research Center, Tehran University of Medical Science, Tehran, Iran; ^2^Rheumatology Research Center, Tehran University of Medical Sciences, Tehran, Iran; ^3^Development of Research and Technology Center, Deputy of Research and Technology, Ministry of Health and Medical Education, Tehran, Iran; ^4^Non-Communicable Diseases Research Center, Endocrinology and Metabolism Population Sciences Institute, Tehran University of Medical Sciences, Tehran, Iran; ^5^Endocrinology and Metabolism Research Center, Endocrinology and Metabolism Clinical Sciences Institute, Tehran University of Medical Sciences, Tehran, Iran; ^6^Hematology, Oncology and Stem Cell Transplantation Research Center, Research Institute for Oncology, Hematology and Cell Therapy, Tehran University of Medical Sciences, Tehran, Iran; ^7^Digestive Diseases Research Center, Digestive Diseases Research Institute, Tehran University of Medical Sciences, Tehran, Iran; ^8^Department of Mathematics, Yasouj University, Yasouj, Iran; ^9^Inflammation Research Center, Tehran University of Medical Sciences, Tehran, Iran

**Keywords:** osteoarthritis, antioxidant, systematic reviews, meta-analysis, cartilage damage, knee osteoarthritis (KOA)

## Abstract

**Objective:**

Knee osteoarthritis (KOA) is one of the growing health problems with a considerable burden. With recent research on the possible effectiveness of antioxidants in the remission of KOA symptoms, a systematic review and meta-analysis was required to confirm this hypothesis.

**Design:**

Literature studies were searched on the most comprehensive databases such as PubMed, International Scientific Indexing, and Scopus, with no language and time restrictions. On 17 July 2021, a search strategy was developed based on the roots of “osteoarthritis (OA)” and “antioxidants,” with no time or language limitations. As the primary outcome, pain was evaluated based on all indicators for evaluating pain [e.g., Western Ontario and McMaster Universities Osteoarthritis Index (WOMAC) pain scores, the visual analog scale (VAS), and the numerical rating scale (NRS)]. The symptoms and functions of KOA and quality of life (QOL) were also considered as secondary outcomes, each of which was measured and reported by the corresponding instrument in the studies. To measure the changes in pain, symptoms, and functions of participants, we included randomized controlled trials with a placebo control or other medical therapeutic interventions. Publication bias was assessed using Begg's funnel plot and Egger's regression test, which was deemed to be statistically significant at 0.1, and the results were checked by the trim-and-fill test.

**Results:**

After refinement, data were extracted from 31 documents from 7,698 primary searched papers. Using the VAS as a reliable psychometric measuring instrument, the present study revealed that a significant difference in the characteristics of disease-related symptoms of patients with KOA was reached after antioxidant therapy (standardized mean difference (SMD): 0.467, 95% confidence interval (CI): 0.303–0.632, *p* < 0.0001). The results reported by WOMAC confirmed no significant difference in the combined score, difficulty score, pain score, and stiffness score.

**Conclusion:**

As the first comprehensive systematic review of the association between antioxidant supplementation and KOA, this study showed that antioxidants can decrease disease-related symptoms in patients with KOA. The results can be useful for health policy decisions and future related studies.

**Systematic review registration:**

https://www.crd.york.ac.uk/prospero/display_record.php?ID=CRD42022351060, identifier: CRD42022351060.

## Introduction

Knee osteoarthritis (KOA) is one of the most common causes of pain, loss of function, and disability, which progressively affects millions of people worldwide and makes their lives difficult (1, 2). This condition caused the protective cartilage that protects the ends of the bone from impact to break down. Evidence suggests a higher incidence of knee degeneration among the elderly and women (1, 3, 4).

According to recent estimates, as a consequence of aging and many other predisposing factors, the global burden of KOA becomes a major health problem. The increasing prevalence rate, economic burden, and adverse health outcomes on the quality of life (QOL) make osteoarthritis (OA) an urgent public health issue (2, 5). Considering the importance and priority of the problem, prevention approaches must be planned and followed as the first line of intervention. However, the successful management and control of the disease will depend on the recognition of early symptoms, accurate diagnosis, and appropriate treatment (5–7).

Much of the research is based on pharmacological, mechanical, and surgical interventions. Therapeutic approaches focus primarily on controlling pain and other symptoms, improving functional abilities, and reducing disease progression (3, 4, 8, 9).

Most treatments also include a combination of regular physical activities, weight control, joint protection, and medication. In severe cases where one of the abovementioned treatments is not successful, surgical methods are recommended (2, 10).

From pharmacological approaches, analgesics and anti-inflammatory compounds were used to reduce inflammation and pain. Antioxidants have been discussed as an alternative treatment in elderly people who are at increased risk of OA and tend to have a poor physical function (3–5). Recent research suggests that some therapeutic approaches have focused on the use of antioxidants to prevent the damage caused by OA to the cartilage. Conflicting evidence has been provided on the effects of antioxidants on disease mortality and morbidity, which still requires further study. Antioxidant supplements should be considered medicinal products and should be adequately evaluated before marketing (3, 6, 11). These studies have been carried out by changing the diet to increase the proportion of foods containing antioxidants or by prescribing antioxidant pharmaceutical forms and supplements (10). Several studies reported improvement and increase in muscle strength, the promotion of physical functions, and a decreased risk of disease progression. They may be rooted in the free radical theory of aging, which hypothesizes that oxygen-derived free radicals are responsible for age-related damages at the cellular and tissue levels (3, 12–14).

In light of this problem's importance, we need more evidence to assist policymakers and clinicians in designing effective interventions. To provide reliable high-level evidence, the present study was designed and conducted as a comprehensive systematic review to assess the effects of antioxidants on KOA.

## Materials and methods

With an aim to assess the association of antioxidants and OA symptoms, we followed the Preferred Reporting Items for Systematic Reviews and Meta-Analyses (PRISMA) statement (15). The review protocol was registered in the PROSPERO international prospective register of systematic reviews (CRD42022351060).

### Data sources and search strategies

Using the Medical Subject Headings (MeSH) terms and Emtree, related published scientific papers and peer review documents were systemically searched on PubMed, ISI/WOS, and Scopus.

On 17 July 2021, a search strategy was developed based on the roots of “OA” and “antioxidants” with no time or language limitations. In case of encountering any non-English articles, we consulted with the Department of Foreign Languages at our university (Tehran University of Medical Sciences) and asked the help of relevant translation experts. Fortunately, no non-English articles entered the final stage of assessment. The results were limited to human subjects. Additional searches were considered for the reference list of studies (Supplementary Table S1).

### Data management and study selection

The searched records were exported to the Endnote software. After three steps of relevance assessment based on titles, abstracts, and full texts, before data extraction, relevant papers were evaluated for their quality. Two studies independently followed all processes. A third reviewer resolved any disagreements between the reviewers.

### Inclusion and exclusion criteria

In the present study, only randomized controlled trials (RCTs) were included.

The results were presented in a PRISMA flowchart according to the PRISMA guideline (16).

The specifically targeted points as inclusion criteria were as follows:

(1) Targeted patients with KOA.

(2) Conducted therapeutic intervention with any kind of antioxidant compared to the placebo or any other medical treatments.

(3) Addressed primary or secondary targeted outcomes.

#### Primary outcomes

All indicators for evaluating pain [e.g., Western Ontario and McMaster Universities Osteoarthritis Index (WOMAC) pain scores, the visual analog scale (VAS), and the numerical rating scale (NRS)].

#### Secondary outcomes

Indicators for evaluating the symptoms and functions of KOA as a whole (e.g., indicators for WOMAC total scores and Lequesne's index) and patient QOL such as the Euro-QoL instrument (EQ5D) and the 36-Item Short-Form Health Survey (SF-36).

If there was more than one paper from the specific unique research, more complete data were considered. Papers with duplicate citations were deleted.

### Quality assessment and data extraction

After three steps of relevance refinement through the Endnote software, including titles, abstracts, and full-text review, using the PRISMA 2020 checklist and Cochrane collaborators tools for assessing the risk of bias in randomized trials (Supplementary Table S2) (17). The quality assessment of the studies was followed by two independent researchers.

Using a predefined checklist, data were extracted from the qualified eligible papers for citation, publication year, study year, the place of study, the type of study, population, total sample size, mean age, the outcomes of interest in pain, symptoms and functions of participants, the type of measure, the results of measures, and other information.

Data extraction and quality assessment were carried out independently by two researchers (kappa statistic for agreement for quality assessment; 0.94). The probable discrepancy was resolved by referring to the opinion of a third expert.

### Data synthesis and meta-analysis

We chose the standardized mean difference (SMD)/Cohen's *d* as the effect size, and eligible studies were meta-analyzed (18).

For the two groups (treatment and placebo), the SMD was calculated using the mean differences (endpoint from baseline) and standard deviations (SD) according to the following formula (19).


$$\begin{array}{l} \text{SMD}=(\text{mean difference for the interventiongroup}-\\ \text{ mean difference for thecontrol group})/\text{pooled SD}\\ \;Pooled\;SD=\;\surd ({\left(SD\;in\;the\;intervention\;group\right)}^{2}\\ \;+\;{\left(SD\;in\;the\;control\;group\right)}^{2})/2). \end{array}$$

A random-effect model was applied if the level of significance of the *Q*-statistic for heterogeneity was set at 0.1 or (*I*^2^ < 50% considered as a fixed effect and *I*^2^ ≥ 50% considered as a random effect) (20).

In other cases, a fixed-effect model was used (21). All analyses were done using Stata version 16 (22). A *p-*value ≤ 0.05 was regarded as statistically significant.

#### Heterogeneity and the risk of bias assessment

The Cochrane *Q* test and *I*^2^ statistic were applied to measure the heterogeneity between the studies (23). The *I*^2^ statistic was used to quantify the degree of heterogeneity between the studies, with *I*^2^ values of 25, 50, and 75% being considered to correspond to low, medium, and high levels of heterogeneity, respectively. The result of the *Q*-test was considered to be statistically significant at 0.1. Publication bias was assessed using Begg's funnel plot and Egger's regression test, which was deemed statistically significant at 0.1, and the results were checked using the trim-and-fill test.

#### Outlier detection and sensitivity analysis

To assess the potential sources of heterogeneity in the results, the Galbraith plot and sensitivity analysis were carried out. Galbraith plot analysis was performed to detect the outliers as the potential sources of heterogeneity (24). In addition, sensitivity analysis, which eliminates any single study at a time, was used to assess the effect of each study on the overall results of a meta-analysis to show the stability and robustness of the results (25).

### Ethical considerations

The present study was approved by the ethics committee of Tehran University of Medical Science (code: IR.TUMS.DDRI.REC.1400.034). All studies included in our review would be cited in all reports and all publications extracted from our study. For further information required, the corresponding authors were contacted.

## Results

### Study selection

**Figure 1:**
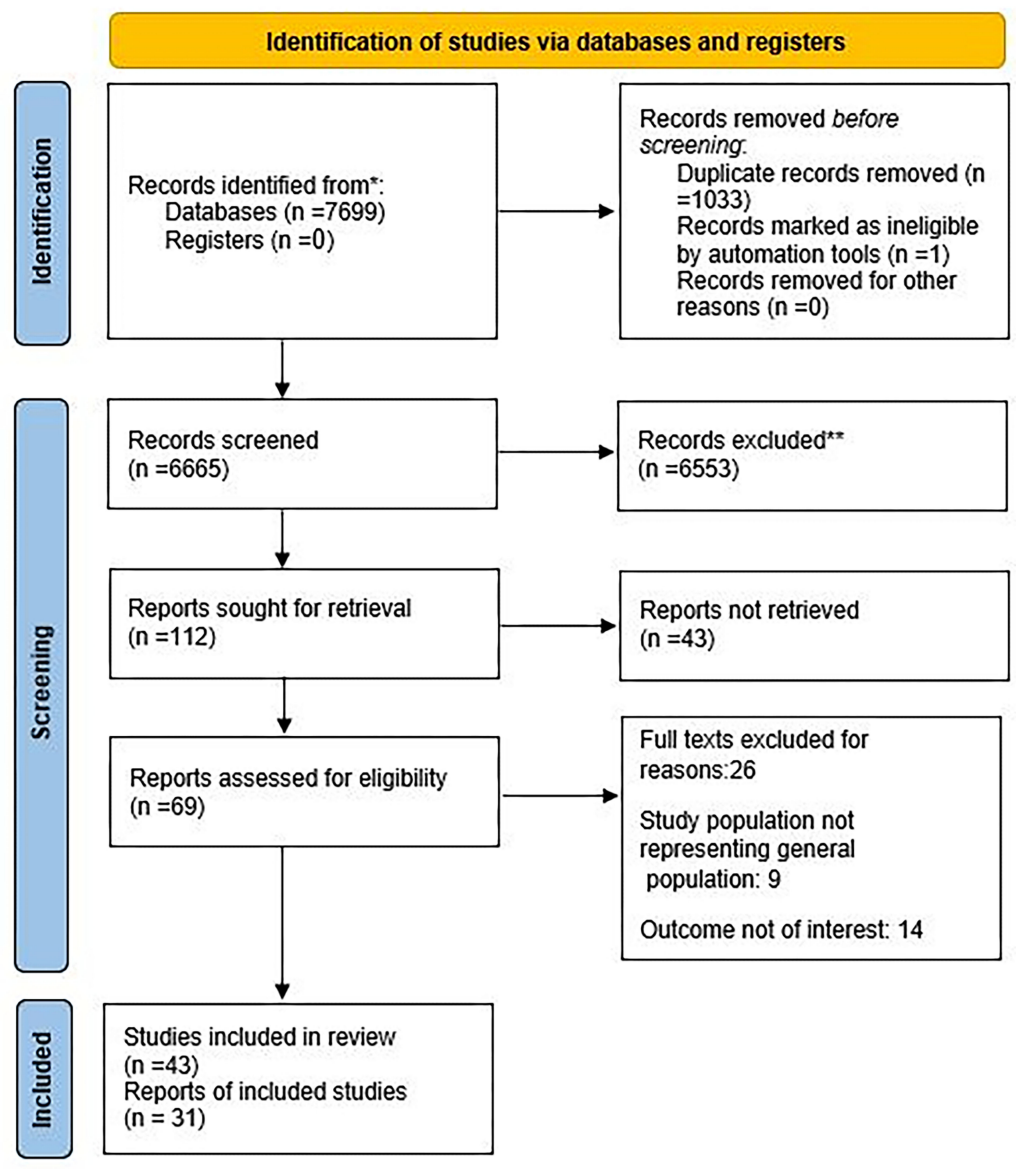
The review flowchart for the selection of the primary studies.

Through a comprehensive search on the targeted databases, we found 7,698 papers. Of these, 1,033 papers were duplicated in different search sources and were excluded. The remaining 6,665 papers were screened according to the relevance of their titles and abstracts. After these refinement steps, 69 papers were left for a full-text review. After relevance assessment and quality control, data were extracted from 31 documents (43 comparison bands) (Figure 1). The kappa statistic for the agreement of processes, between the two independent research experts, from the search study development to data extraction and analysis was 0.94, which showed a good agreement.

### Characteristics of the primary studies

We included 43 trial studies and pooled the results by including 6,605 participants in the data analysis. Of these, 10 studies were designed as multicenter RCTs.

Ten studies were designed as multi-center RCTs as the most significant number of studies performed; 10 articles were carried out in China, 6 in Iran, and 2 in Japan, Italy, France, the US, and Belgium. From other countries, only one paper was included in the study.

The first analytic studies were conducted in 1998, and the last paper was from 2020. Considering a variety of methodological approaches to the included studies, our systematic review yielded different measures of the association including hazard ratio (HR), odds ratio (OR), and relative risk (RR). Six papers did not mention the sex distribution of their participants, and two papers did not provide information on the age of participants. The characteristics of the included studies are presented in Table 1.

**Table 1:** The characteristics of included papers.

**NO**	**Citation**	**Country**	**Scope**	**Study year**	**Total sample size**	**Intervention sample Size**	**Control sample Size**	**Intervention group sex rate**	**Control group sex rate**	**Mean age of intervention group**	**Mean age of control group**	**Type of intervention**	**Duration of intervention**	**Out comes measure**	**Main conclusion**
1	Bolognesi et al. (26)	China	Single-center	2018	120	60	60	0.417	0.383	60.4	61.3	Celecoxib + glucosamine sulfate / Celecoxib	8 weeks	VAS, WOMAC	The combination of celecoxib with glucosamine sulfate effectively reduces immune inflammatory response, and joint pain associated with KOA
2	Srivastavs et al. (27)	India	Single-center	-	160	78	82	0.679	0.610	50.23	50.27	CL extract/ Placebo	120 days	VAS, WOMAC	Clinically, the VAS and WOMAC scores became Better
3	Vishal et al. (28)	India	Single-center	2011	60	30	29	0.633	0.621	53.2	55.3	Aflapin/ Placebo	NM	VAS, WOMAC	The changes were not significant
4	Kanzaki al. (29)	Japan	Single-center	2008	40	20	20	0.800	0.850	55.1	58.3	glucosamine hydrochloride, chondroitin/ placebo	16 weeks	VAS	GCQ supplement was more effective than placebo in decreasing the KOA-associated clinical symptoms
5	Velangi et al. (30)	India	Single-center	-	100	50	50	0.500	0.500	-	-	Vitamin D3 + Virgin Coconut Oil/ Vitamin D3	12 weeks	VAS, WOMAC	The mean difference of effect in all parameters was higher in intervention group than control group
6	Kuptniratsaikul et al. (31)	Thailand	Multi-center	-	367	171	160	0.918	0.869	60.3	60.9	C. domestica extracts / Ibuprofen	4 weeks	WOMAC	C. domestica extracts are as effective as ibuprofen for the treatment of KOA
7	Pavelka et al. (32)	CZ, SK, HU, PL, RO	Multi-center	-	366	142	121			62.3	62.2	Piascledine^®^ 300 mg/ chondroitin sulfate	180 days	WOMAC	The first direct comparison between avocado soybean unsaponifiable 300 mg once daily and chondroitin sulfate three times daily revealed no difference in efficacy or safety aspects
8	Debbi et al. (33)	Israel	Single-center	2006	59	25	25	0.840	0.480	67	71	Methylsulfonylmethane/ placebo	12 weeks	VAS, WOMAC	Patients in intervention group showed small clinical improvement in pain and physical function
9	Kanzak, et al. (34)	Japan	Single-center	2012	100	50	50	0.560	0.560	51.9	51.6	gCQID, glucosamine hydrochloride, chondroitin sulfate, type II collagen peptides, quercetin glycosides, imidazole peptides, and vitamin D/ placebo	16weeks	VAS	GCQID supplementation was safe and effective in improving knee-joint functions and locomotor functions in subjects with knee pain
10	Notarnicol et al. (35)	Italy	Single-center	-	120	54	58	0.704	0.724	58.7	59.5	MEBAGA (Methylsulfonylmethane and Boswellic Acids versus Glucosamine sulfate in the treatment of knee Arthritis)/ Glucosamine sulfate	3 months	VAS	This new combination of integration (MSM and BS) has good results in comparison with GS, until now the cornerstone of the treatment of arthritis in according to guidelines
11	McAlindon et al. (36)	Italy	Na (registry based)	-	54	28	26	0.357	0.385	52.3	53.1	Movardol^®^, Leonardo Medica, Vinci, Florence, Italy (N-acetyl-D-glucosamine, Ginger, Boswellia, boswellic acids) / standard management	6 months	WOMAC	Oral supplementation with a combination of products of natural origin can be effectively used to target multiple OA signs and symptoms
12	Farid et al. (37)	Iran	Single-center	-	40	17	16	0.706	0.813	55	49.71	Pycnogenol pills/ placebo	3 months	WOMAC	The efficacy of Pycnogenol detected in alleviating osteoarthritis symptoms and reducing the need for NSAIDs
13	Jacquet et al. (13)	France	Single-center	-	81	41	40	0.659	0.700	56.8	57.5	fish oils rich in omega-3 and omega-6 fatty acids, Urtica dioica (the common nettle)/ zinc and vitamin E; or an identical placebo	3 months	WOMAC	Use of three capsules a day over three months of this nutraceutical com-pound might decrease disease scores in patients KOA
14	Farid et al. (38)	Iran	Single-center	-	40	19	18	0.947	0.944	47.5	48.9	Pycnogenol pills/ placebo	3 months	WOMAC	Alleviating KOA symptoms and reducing the need for NSAIDs or COX-2 inhibitors Administration detected
15	Panda et al. (39)	India	Single-center	2017	50	25	25	-	-	55.2	53.12	Curene/ placebo	60 days	VAS, WOMAC	This study validates a potential application of Curene as a useful alternative treatment option for the symptoms of KOA
16	Henrotin et al. (40)	India	Single-center	2006	120	29	24	0.552	0.792	56.63	56.8	NR-INF-02/ glucosamine sulfate (GS)	42 days	VAS, WOMAC	This study effectively demonstrated acceptable efficacy and tolerability profile of NR-INF-02 in a small group of subjects indicating its utility as one of the treatment options in symptomatic management of pain among patients with uncomplicated knee OA
17	Henrotin et al. (40)	India	Single-center	2006	120	29	28	0.552	0.786	56.63	58.17	NR-INF-02/ NR-INF-02 and GS	42 days	VAS, WOMAC	
18	Henrotin et al. (40)	India	Single-center	2006	120	29	29	0.552	0.552	56.63	56.77	NR-INF-02/ placebo	42 days	VAS, WOMAC	
19	Atabaki et al. (8)	Iran	Single-center	-	30	15	15	-	-	49.13	48.26	Curcumin / placebo	3 months	VAS	The curcumin can significantly reduce inflammation and pain in patients with O
20	Maheu et al. (41)	France	Multi-center	1994	164	85	79	0.741	0.696	63.3	65.1	ASU (avocadoisoybean unsaponifiables) / placebo	6 months	VAS	Significant symptomatic efficacy over placebo in the treatment of OA, acting from month 2 and showing a persistent effect after the end of treatment
21	Madhu et al. (42)	US	Single-center	2009	146	73	73	0.671	0.548	61.8	63	Vitamin D Supplementation / placebo	24 months	WOMAC	Vitamin D compared with placebo, did not reduce knee pain or cartilage volume loss in patients with symptomatic KOA
22	Panahi et al. (43)	Iran	Single-center	2012	53	27	26	0.667	0.846	57.32	57.57	Curcuminoids / placebo	6 months	WOMAC	Curcuminoids represent an effective and safe alternative treatment for KOA
23	Altman et al. (44)	US	Multi-center	-	261	124	123	59.700	63.400	64.06	66.36	Ginger Extract /placebo	6 weeks	WOMAC	A highly purified and standardized ginger extract had a statistically significant effect on reducing symptoms of KOA. This effect was moderate
24	Appelbo et al. (45)	Belgium	Multi-center	-	260	86	86	0.651	-	63.4	65.2	ASU 300/ ASU 600	3 moths	VAS	The efficacy of ASU at a dosage of 300mg/day and 600mg/day was consistently superior to that of placebo at all endpoints, with no differences observed between the two doses
25	Appelbo et al. (45)	Belgium	Multi-center	-	260	86	88	0.849	-	63.4	66.3	ASU 300/ Placebo	3 moths	VAS	
26	Kimmatkar et al. (46)	India	Single-center	-	62	31	31	-	-	-	-	RA- 11	32 weeks	VAS, WOMAC	This controlled drug trial demonstrates the potential efficacy and safety of RA- 11 in the symptomatic treatment of OA knees over 32 weeks of therapy
27	Pavelka et al. (32)	India	Single-center	2013	91	29	30	0.517	0.500	43.7	45.1	NXT15906F6 (TamaFlexTM) 250 mg/ NXT15906F6 (TamaFlexTM) 400mg	90 days	WOMAC	NXT15906F6 provided substantial relief from knee pain after physical activity and improved joint function in non-arthritic adults
28	Pavelka et al. (32)	India	Single-center	2013	91	29	30	0.517	0.500	43.7	45.3	NXT15906F6 (TamaFlexTM) 250 my/ placebo	90 days	WOMAC	
29	Dehghan et al. (47)	Iran	Single-center	2014	120	35	38	0.629	0.553	47.54	46.78	oral diclofenac + oral vitamin E/ oral diclofenac + oral B vitamin	21 days	VAS, WOMAC	The mean score of WOMAC and VASs of knee pain, total pain severity, knee joint stiffness, and function decreased significantly two groups
30	Dehghan et al. (47)	Iran	Single-center	2014	120	35	37	0.629	0.541	47.54	46.4	oral diclofenac + oral vitamin E/ oral diclofenac + placebo	21 days	VAS, WOMAC	
31	Rao et al. (48)	India	Single-center	2008	60	19	19	0.842	0.632	51.6	53.2	5-Loxin/ Aflapin	90 days	VAS, WOMAC	Aflapin exhibited better efficacy compared to 5-Loxin^®^. In comparison with placebo, the safety parameters were almost unchanged in the treatment groups
32	Rao et al. (48)	India	Single-center	2008	60	19	19	0.842	0.526	51.6	52.4	5-Loxin/ Placebo	90 days	VAS, WOMAC	
33	Lubis et al. (49)	Indonesia	Single-center	-	147	49	50	65.300	78.000	60.9	58.3	glucosamine-chondroitin sulfate/ glucosamine-chondroitin sulfate-methylsulfonylmethane	3 months	VAS, WOMAC	There were significant differences between three treatment groups on the WOMAC and VAS scores
34	Lubis et al. (49)	Indonesia	Single-center	-	147	49	48	65.300	41.700	60.9	62.8	glucosamine-chondroitin sulfate/ placebo	3 months	VAS, WOMAC,	
35	Sengupta et al. (7)	India	Single-center	-	75	24	23	0.708	0.652	52.37	53.22	5-Loxin/ Aflapin	90 days	WOMAC	Aflapin exhibited better efficacy compared to 5-Loxin^®^
36	Sengupta et al. (7)	India	Single-center	-	75	24	23	0.708	0.783	52.37	52.43	5-Loxin/ placebo	90 days	WOMAC	In comparison with placebo, the safety parameters were almost unchanged in the treatment groups
37	Haroyan et al. (50)	Armenia	Multi-center	2016	210	66	67	0.909	0.925	54.65	57.91	CuraMed^®^ 500-mg capsules (333 mg curcuminoids)/ Curamin^®^ 500-mg capsules (350 mg curcuminoids and 150 mg boswellic acid)	12 weeks	WOMAC	Favorable effects of both preparations compared to placebo were observed after only 3 months of continuous treatment
38	Haroyan et al. (50)	Armenia	Multi-center	2016	210	66	68	0.909	0.956	54.65	56.04	CuraMed^®^ 500-mg capsules (333 mg curcuminoids)/ placebo	12 weeks	WOMAC	A significant effect of Curamin^®^ compared to placebo was observed both in physical performance tests and the WOMAC joint pain index
39	Henrotin et al. (40)	Belgium	Multi-center	2017	150	32	33	85.100	79.600	61.4	60.9	bio-optimized Curcuma longa extracts (BCL) low dosage/ BCL high dosage	3-month	VAS	daily intake of 186.6 mg/day of BCL in patients with symptomatic knee OA leads to a reduction in pain
40	Henrotin et al. (40)	Belgium	Multi-center	2017	150	32	36	85.100	75.600	61.4	63.3	bio-optimized Curcuma longa extracts (BCL) low dosage/ placebo	3-month	VAS	BCL is superior to placebo with a good safety profile and a good compliance
41	Chopra et al. (51)	India	Single-center	2007	440	75	86	_	_	55.55	55.28	Ayurvedic formulations (SGCG)/ SGC	24 weeks	VAS, WOMAC	Ayurvedic formulations (especially SGCG) significantly reduced knee pain and improved knee function
42	Chopra et al. (51)	India	Single-center	2007	440	75	75	_	_	55.55	55.51	Ayurvedic formulations (SGCG)SGCG/ glucosamine sulfate	24 weeks	VAS, WOMAC	Ayurvedic formulations (especially SGCG) were equivalent to glucosamine
43	Chopra et al. (51)	India	Single-center	2007	440	75	78	_	_	55.55	56.6	Ayurvedic formulation (SGCG)SGCG/ celecoxib	24 weeks	VAS, WOMAC	Ayurvedic formulations (especially SGCG) were equivalent to celecoxib

### Results of meta-analyses

#### The effects of antioxidants on KOA: The VAS

A random-effect meta-analysis of 27 trials and the pooled results obtained from 2,394 participants (1,193 cases and 1,201 controls) showed no significant difference in the VAS after antioxidant therapy (SMD: 0.045, 95% confidence interval (CI): (−0.78–0.87), *p* = 0.91) (Supplementary Figure S1). There was high heterogeneity between studies (*p* < 0.0001; *I*^2^ = 98.78%). Egger's test provided no evidence of publication bias (Egger's regression intercept: −3.13, 95%CI: (−10.90–4.64), *p* = 0.414). Begg's funnel plot of standard error (SE) vs. effect size (SMD) was asymmetric (Supplementary Figure S2). The Galbraith plot demonstrated that 15 studies were outside the 95% CI, which means they are outliers (Supplementary Figure S3).

**Figure 2:**
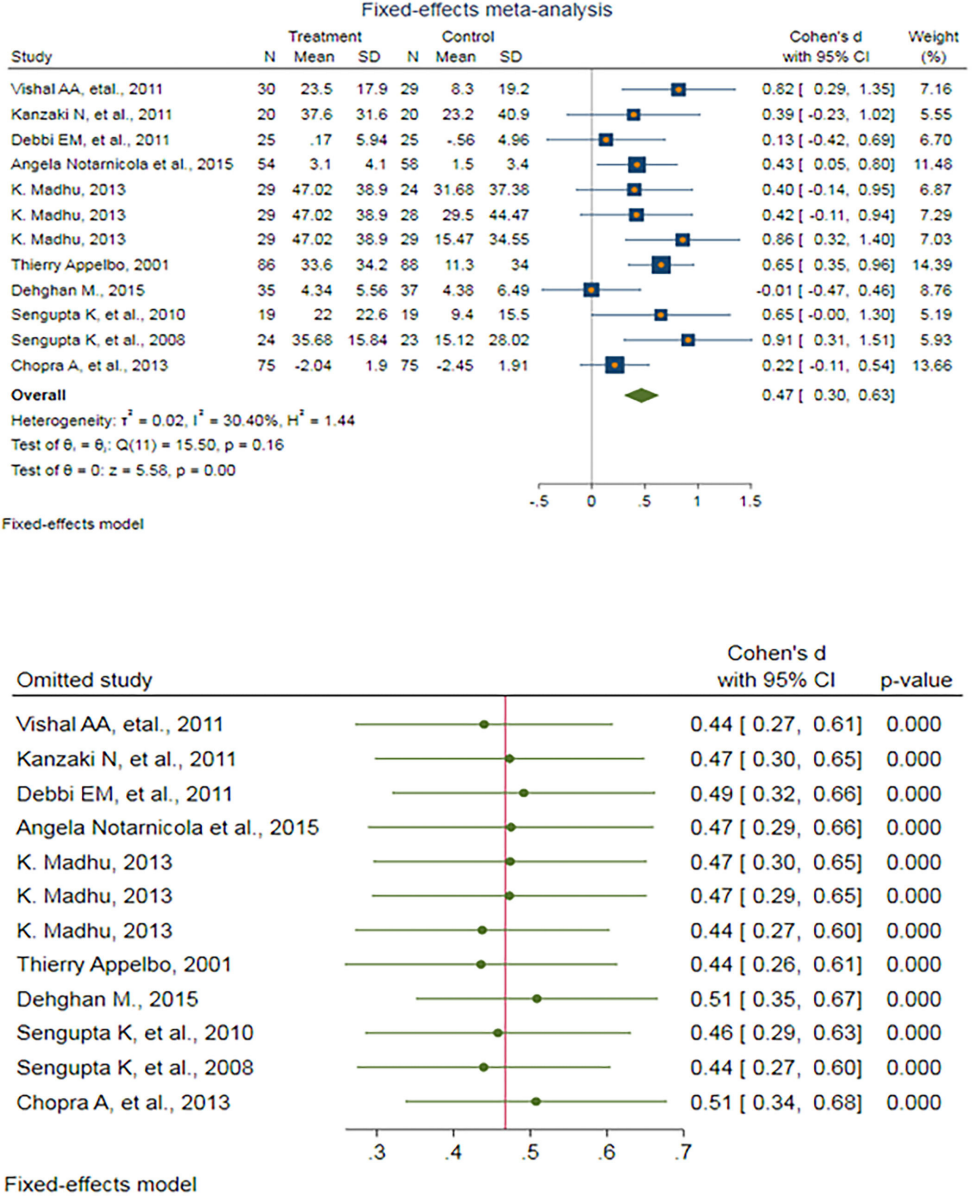
**(UP)** A forest plot for the association between antioxidants and the outcomes of interest in osteoarthritis (OA) based on the visual analog scale (VAS). **(DOWN)** Forest plot of sensitivity analysis for the association between antioxidants and the outcomes of interest in OA based on the VAS.

After eliminating outlier studies, a fixed-effect meta-analysis of 12 trials and the pooled results obtained by including 910 participants (455 cases and 455 controls) showed no significant difference in the VAS after antioxidant therapy (SMD: 0.467, 95%CI: (0.303–0.632), *p* < 0.0001) (Figure 2). There was low heterogeneity between the studies (*p* = 0.161; *I*^2^ = 30.40%), which is not statistically significant. Egger's test provided no evidence of any publication bias (Egger's regression intercept: 0.802, 95%CI: (−2.25 3.85), *p* = 0.571). Begg's funnel plot of SE vs. effect size (SMD) was symmetrical, and the trim-and-fill correction also suggested no potentially missing studies on any side of the funnel plot (Supplementary Figure S4).

Sensitivity analyses were performed to test the robustness of the observed association. The elimination of any single study at a time from the meta-analysis ranged from 0.44 (95%CI: 0.26–0.61) to 0.51 (95%CI: 0.35–0.67), which indicated a high robustness of the pooled estimates of prevalence (Figure 2).

#### The effects of antioxidants on KOA: WOMAC combined pain score

A random-effect meta-analysis of 22 trials and the pooled results obtained by including 2,378 participants (1,204 cases and 1,174 controls) showed no significant difference in the WOMAC combined pain score after antioxidant therapy (SMD: 0.117, 95%CI: (−0.319–0.554), *p* = 0.60) (Supplementary Figure S5). There was high heterogeneity between the studies (*p* < 0.0001; *I*^2^ = 96.18%). Egger's test did not provide any evidence of publication bias (Egger's regression intercept: −0.55, 95%CI: (−5.85–4.75), *p* = 0.831). Begg's funnel plot of SE vs. effect size (SMD) was asymmetrical (Supplementary Figure S6).

The Galbraith plot revealed that eight studies were outside the 95% CI, which means that they are outliers (Supplementary Figure S7).

**Figure 3:**
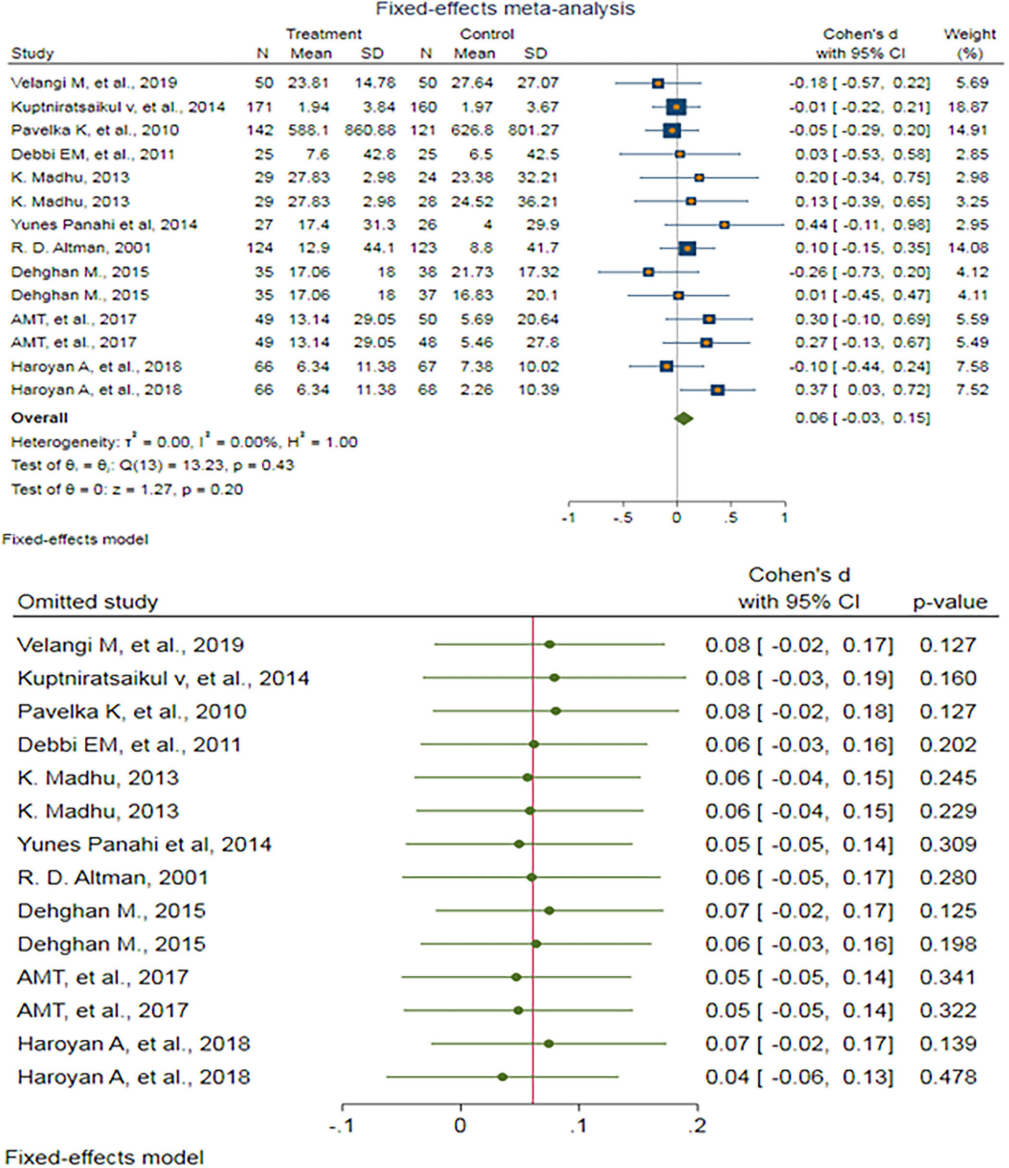
**(UP)** A forest plot for the association between antioxidants and the outcomes of interest in OA based on the Western Ontario and McMaster Universities Osteoarthritis Index (WOMAC) combined pain score. **(DOWN)** Forest plot of sensitivity analysis for the association between antioxidants and the outcomes of interest in OA based on the WOMAC combined pain score.

After removing outlier studies, a fixed-effect meta-analysis of 14 trials and the pooled results obtained by including 1,762 participants (897 cases and 865 controls) showed no significant difference in the WOMAC combined pain score after antioxidant therapy (SMD: 0.061, 95%CI: −0.033–0.154) *p* = 0.203) (Figure 3). There was no heterogeneity between the studies (*p* = 0.43; *I*^2^ = 0.00%). Egger's test provided no evidence of publication bias (Egger's regression intercept: 0.81, 95%CI: (−1.08–2.70), *p* = 0.369). Begg's funnel plot of SE vs. effect size (SMD) was asymmetrical (Supplementary Figure S8). The trim-and-fill correction suggested two potentially missing studies on the left side of the funnel plot (Supplementary Figure S9). Imputation for these potentially missing studies yielded an effect size of 0.025 (95%CI: −0.080–0.130), which was not statistically significant.

Sensitivity analyses were done to test the robustness of the observed association. The elimination of any single study at a time from the meta-analysis ranged from 0.04 (95%CI: −0.06–0.13) to 0.08 (95%CI: −0.02–0.18), indicating high robustness of the pooled estimates of prevalence (Figure 3).

#### The effects of antioxidants on KOA: WOMAC difficulty pain score

A random-effect meta-analysis of 25 trials and the pooled results obtained by including 2,547 participants (1,285 cases and 1,262 controls) showed no significant difference in the WOMAC difficulty pain score after antioxidant therapy (SMD: 0.296, 95%CI: −0.277–0.869), *p* = 0.31) (Supplementary Figure S10). There was non-ignorable heterogeneity between the studies (*p* < 0.0001; *I*^2^ = 97.94%). Egger's test provided no evidence of publication bias (Egger's regression intercept: 1.89, 95%CI: (−1.88–5.67), *p* = 0.310). Begg's funnel plot of SE vs. effect size (SMD) was asymmetrical (Supplementary Figure S11).

The Galbraith plot revealed that 11 studies were outside the 95%CI, which means they are outliers (Supplementary Figure S12).

**Figure 4:**
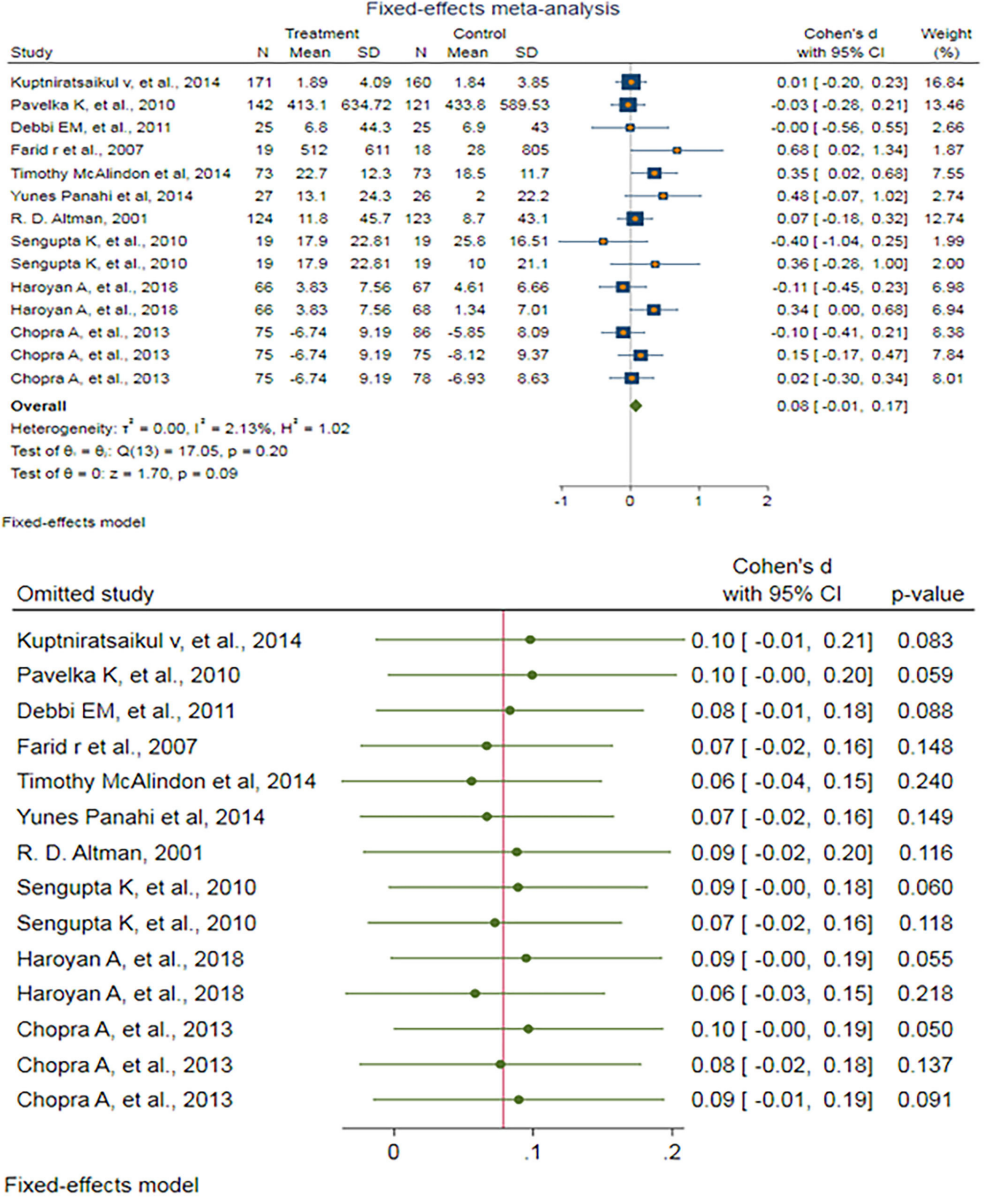
**(UP)** A forest plot for the association between antioxidants and the outcomes of interest in OA based on the WOMAC difficulty pain score. **(DOWN)** Forest plot of sensitivity analysis for the association between antioxidants and the outcomes of interest in OA based on the WOMAC difficulty pain score.

After excluding outlier studies, a fixed-effect meta-analysis of 14 trials and the pooled results obtained by including 1,934 participants (976 cases and 958 controls) showed no significant difference in the WOMAC difficulty pain score after antioxidant therapy (SMD: 0.079, 95%CI: −0.012–0.170), *p* = 0.09) (Figure 4). There was ignorable heterogeneity between the studies (*p* = 0.197; *I*^2^ = 2.13%). Egger's test provided no evidence of publication bias (Egger's regression intercept: 1.16 (95%CI: −0.79–3.12), *p* = 0.219). Begg's funnel plot of SE vs. effect size (SMD) was a bit asymmetric (Supplementary Figure S13). The trim-and-fill correction suggested one potentially missing study on the left side of the funnel plot (Supplementary Figure S14). Imputation for this potentially missing study yielded an effect size of 0.068 (95%CI: −0.024–0.160), which was not statistically significant.

Sensitivity analyses were done to test the robustness of the observed association. The elimination of any single study at a time from the meta-analysis ranged from 0.06 (95%CI: −0.04–0.15) to 0.10 (95%CI: −0.01–0.21), indicating high robustness of the pooled estimates of prevalence (Figure 4).

#### The effects of antioxidants on KOA: WOMAC pain score

A random-effect meta-analysis of 28 trials and the pooled results obtained by including 2,840 participants (1,427 cases and 1,413 controls) showed no significant difference in the WOMAC pain score after antioxidant therapy ((SMD: 0.297, 95%CI: −0.125–0.718), *p* = 0.17) (Supplementary Figure S15). There was high heterogeneity between the studies (*p* < 0.0001; *I*^2^ = 96.61%). Egger's test provided no evidence of publication bias (Egger's regression intercept: 2.09 (95%CI: −1.44–5.63), *p* = 0.235). Begg's funnel plot of SE vs. effect size (SMD) was asymmetrical (Supplementary Figure S16).

The Galbraith plot revealed that 10 studies were outside the 95%CI, which means they are outliers (Supplementary Figure S17).

**Figure 5:**
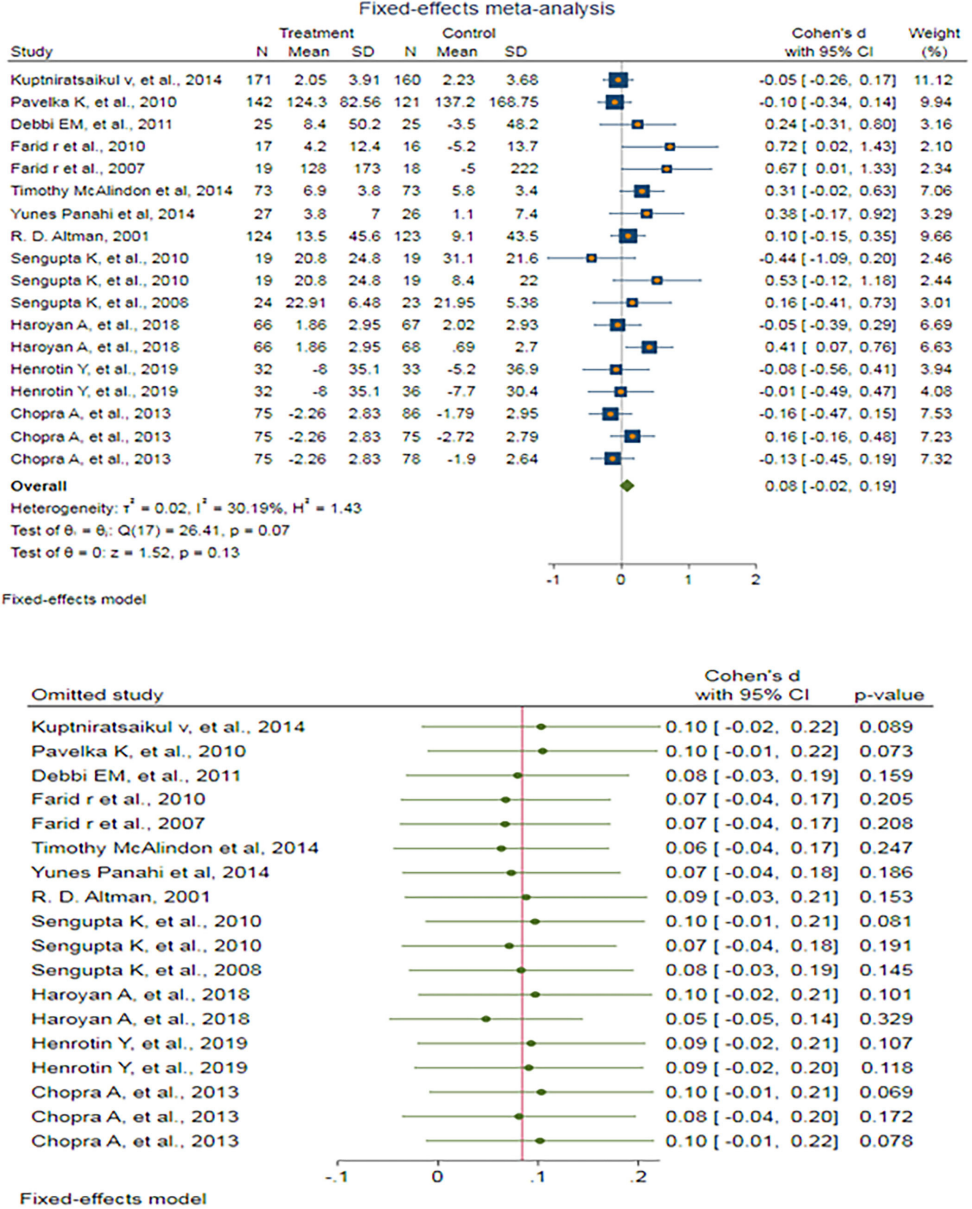
**(UP)** A forest plot for the association between antioxidants and the outcomes of interest in OA based on the WOMAC pain score. **(DOWN)** Forest plot of sensitivity analysis for the association between antioxidants and the outcomes of interest of OA based on the WOMAC pain score.

A fixed-effect meta-analysis of 18 trials and the pooled results obtained by including 2,147 participants (1,081 cases and 1,066 controls) showed no significant difference in the WOMAC pain score after antioxidant therapy (SMD: 0.084 (95%CI: −0.024–0.192), *p* = 0.127) (Figure 5). There was low heterogeneity between the studies (*p* = 0.067; *I*^2^ =30.19%). Egger's test provided no evidence of publication bias (Egger's regression intercept: 1.55 (95%CI:−0.08–3.18), *p* = 0.061). Begg's funnel plot of SE vs. effect size (SMD) was asymmetrical (Supplementary Figure S18). The trim-and-fill correction suggested three potentially missing studies on the left side of the funnel plot (Supplementary Figure S19). Imputation for these potentially missing studies yielded an effect size of 0.044 (95%CI: −0.067–0.155), which was not statistically significant.

Sensitivity analyses were done to test the robustness of the observed association. The elimination of any single study at a time from the meta-analysis ranged from 0.05 (95%CI: −0.05–0.14) to 0.10 (95%CI: −0.02–0.22), indicating high robustness of the pooled estimates of prevalence (Figure 5).

#### The effects of antioxidants on KOA: WOMAC stiffness score

A random-effect meta-analysis of 25 trials and the pooled results obtained by including 2,388 participants (1,208 cases and 1,180 controls) showed no significant decrease in the WOMAC stiffness score after antioxidant therapy (SMD: 0.128 (95%CI: −0.168–0.424), *p* = 0.40) (Supplementary Figure S20). There was high heterogeneity between the studies (*p* < 0.0001; *I*^2^ = 91.79%). Egger's test provided no evidence of publication bias (Egger's regression intercept: 0.64 (95%CI: −2.58–3.87), *p* = 0.685). Begg's funnel plot of SE vs. effect size (SMD) was symmetrical to some extent (Supplementary Figure S21).

The Galbraith plot (Supplementary Figure S22) revealed that seven studies were outside the 95%CI, which means that they are outliers.

**Figure 6:**
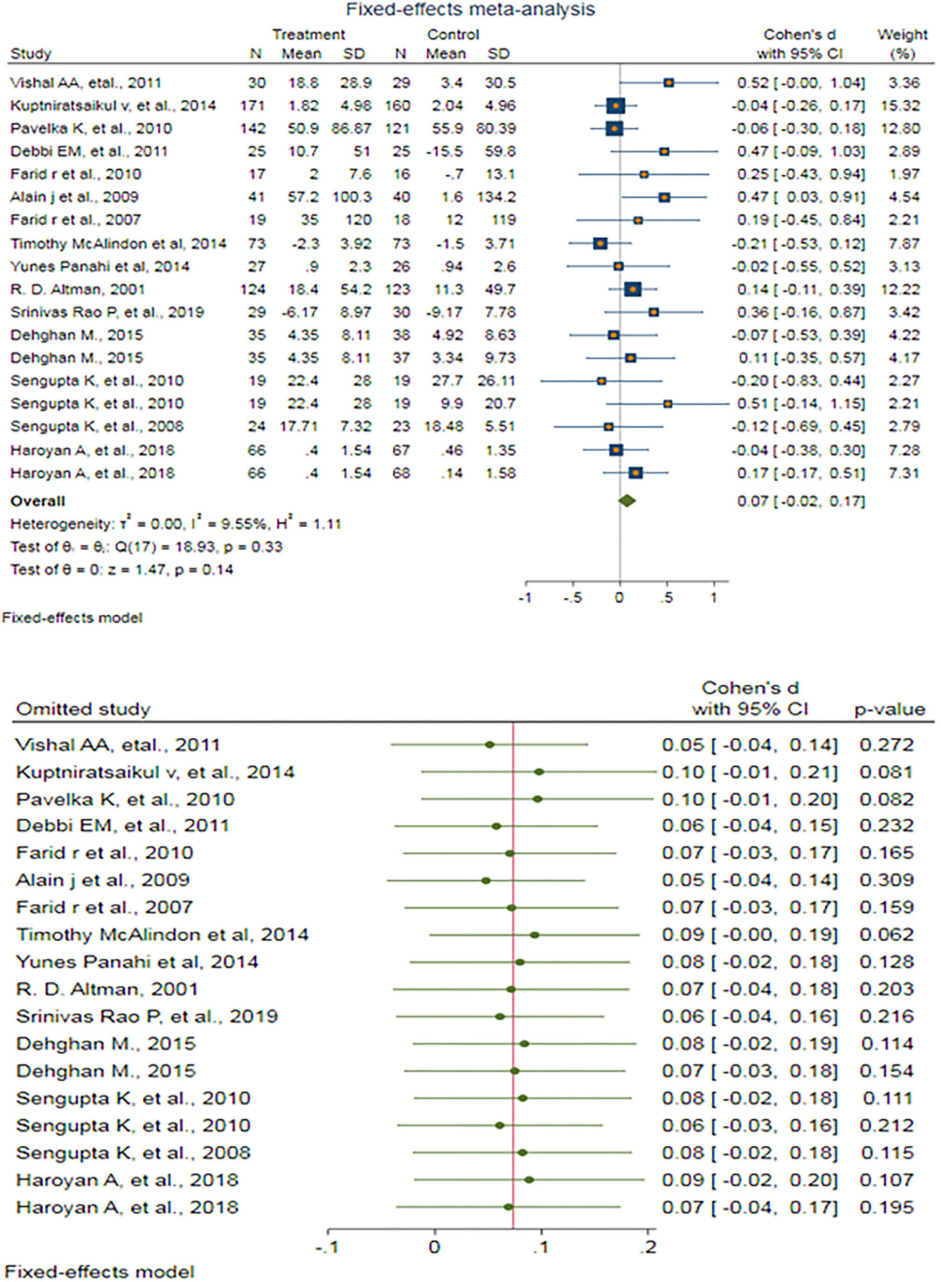
**(UP)** A forest plot for the association between antioxidants and the outcomes of interest in OA based on the WOMAC stiffness score. **(DOWN)** Forest plot of sensitivity analysis for the association between antioxidants and the outcomes of interest in OA based on the WOMAC stiffness score.

A fixed-effect meta-analysis of 18 trials and the pooled results obtained by including 1,894 participants (962 cases and 932 controls) showed no significant decrease in the WOMAC stiffness score after antioxidant therapy (SMD: 0.074 (95%CI: −0.024–0.172), *p* = 0.140) (Figure 6). There was very low and ignorable heterogeneity between the studies (*p* = 0.332; *I*^2^ = 9.55%). Egger's test provided no evidence of publication bias (Egger's regression intercept: 1.27 (95%CI: −0.06–2.60), *p* = 0.06). Begg's funnel plot of SE vs. effect size (SMD) was asymmetrical (Supplementary Figure S23). The trim-and-fill correction suggested five potentially missing studies on any side of the funnel plot (Supplementary Figure S24). Imputation of these potentially missing studies yielded an effect size of −0.001 (95%CI: −0.116–0.113), which was also not statistically significant.

Sensitivity analyses were done to test the robustness of the observed association. The elimination of any single study at a time from the meta-analysis ranged from 0.05 (95% CI: −0.04–0.14) to 0.10 (95%CI: −0.01–0.21), indicating high robustness of the pooled estimates of prevalence (Figure 6).

## Discussion

Our systematic review, as the first comprehensive study of the effects of antioxidants on KOA, provides several major findings. We included 43 trial studies and the pooled results obtained by including 6,605 participants in the data analysis. Of these, 10 studies were designed as multicenter RCTs. A number of substances have been studied in several studies to treat or relieve the signs and symptoms of KOA. Some substances, such as glucosamine, coconut oil, vitamin D, ginger, Boswellia, and vitamin E, were prescribed in the form of compounds or as supplements along with other treatments.

Our findings are in accordance with those of other published RCTs or systematic reviews assessing the role of nutritional compounds or supplements for the prevention and reduction of the symptoms of KOA (52, 53). To compare the intervention effects of different types of antioxidants on the outcomes of interest in OA, we detected significant heterogeneity in the qualified included trials. According to the results detected using VASs as reliable psychometric measuring instruments, the present study revealed a significant difference in the characteristics of disease-related symptoms of patients with KOA who were achieved after antioxidant therapy (*p* < 0.0001). It is worth mentioning that the results reported by the WOMAC confirm no significant difference in the combined score, difficulty score, pain score, and stiffness score. Overall, according to the analysis of the results of standard indicators that measure the clinical symptoms of patients with KOA, it seems that the effectiveness of these compounds mainly prevents, reduces, and relieves symptoms, and studies have shown little success in the treatment and complete relief of clinical symptoms.

In addition to our experience, previous attempts have studied the difficulties and limitations of the problem. Lack of standards for many prescribed compounds, the possibility of the interference of many known and unknown factors on the type and severity of patients' symptoms, scattering of measuring instruments, self-reported baseline data and intervention effects, poorly conducted studies, lack of uniformity in the definition and diagnosis of diseases, and the deficiencies of some papers in the detail of the studies have been mentioned as some determining criteria in the promotion of scientific evidence used in making policies and clinical decisions (1, 3, 4, 32).

The results obtained from related studies are inconsistent on the effectiveness of antioxidants in the control and management of the clinical symptoms of patients with KOA. Much related research has shown that the different components of antioxidants are effective in reducing pain, stiffness, swelling, and improving physical function compared to both placebo or other non-antioxidant interventions (12, 46, 48, 54). The antioxidant supplements with the most evidence of benefit for pain management and function in patients with KOA were based on curcumin, avocado, soya bean, and vitamins D and E (3, 4, 36, 40, 42, 55). Boswellia and some herbs used in Ayurvedic and Chinese medicine may also be useful (14, 26, 46).

In the studies that have addressed this problem, different results have been reported, from the lack of a statistically significant effect of some antioxidant compounds to the registration of some significant indicators in the results of valid measurement tests such as VAS and WOMAC (3, 12–14).

According to the available evidence, the differences in the results obtained from the studies, in addition to what was mentioned above about the designs, implementations, and reporting of results, may be rooted in the complex mechanisms of action of these compounds and various influencing factors (2, 8, 9).

As a possible mechanism of action, antioxidants act against reactive oxygen species, which are produced by cells within the joints and slow down or stop the oxidative damage to various macromolecules and immunomodulatory effects on T- and B-cell functions. These mechanisms have been confirmed through previous *in vivo* and investor studies for vitamin C, vitamin E, and carotenoids (3, 4, 8, 9).

Considering previous attempts, the present study benefits from several achievements. This report presents scientific evidence to depict an association between antioxidants and KOA outcomes. All available sources of related data search use the most comprehensive database of related and most effective systematic search approaches. Despite data dispersion and serious differences in the design and implementation of studies, an attempt was made to present the best practical format of findings using appropriate updated analysis methods based on subgroup and comparative analyses.

Throughout this research, we faced several limitations. As the main limitation, the validity and applicability of the data included in a systematic review depend on the quality of the primary studies. Moreover, the widespread heterogeneity of the searched results limits the generalization of our findings. There was high heterogeneity between the studies that limited the possibility of aggregate analysis and required the elaboration and application of special statistical and analytical techniques. Also, the study design and research interventions were widely dispersed, which were considered in the final analysis. We must select the best approach to address differences in study design and the population that may play a major source of controversy in the analysis. However, the complex and multifactorial nature of antioxidants and possibly unknown interactive mechanisms should be considered in the exploitation of results.

As a practical implication, it could be recommended to increase the intake of antioxidant vitamins in the general diet of OA at-risk groups, especially in susceptible ethnic studies. Meanwhile, the possible effects of high doses of antioxidant vitamins as dietary supplements need to be evaluated against side effects. Many subjects at high risk for KOA and its complications may benefit from a combination of antioxidant and anti-inflammatory compounds. It is also recommended to study the beneficial effects of certain combination protocols for an intervention or for reducing specific symptoms of the disease through well-defined RCTs.

## Conclusion

To our knowledge, this is the first comprehensive systematic review of the association between antioxidant supplementation and OA outcomes. Although the results were not statistically significant, in terms of the reported changes in clinical symptoms and the simplicity and cost-effectiveness of the interventions, it could be recommended to increase the intake of antioxidant vitamins in the general diet of OA at-risk groups, especially in susceptible ethnic studies. The antioxidant supplements with the most evidence of benefit for pain relief and function in KOA were based on curcumin. The benefits of diets containing appropriate antioxidants should be assessed because they may be more economical and easier to incorporate into the lifestyle. The results could be useful for better health policy decisions and future studies in this field. They can also be used for complementary analyses in the future.

## Data availability statement

The original contributions presented in the study are included in the article/Supplementary material, further inquiries can be directed to the corresponding authors.

## Author contributions

MN, SD, and AK wrote this manuscript. SD, AK, and STF contributed to the study design and the interpretation of the results. A-HM and MN were responsible for literature review and data extraction. AK performed the whole statistical analysis. All authors read and approved the final manuscript.
