# Supplementary material for: The effects of antioxidants on knee osteoarthritis: A systematic review and meta-analysis
Source: Front Nutr. 2022 Dec 19;9:1026450. doi: 10.3389/fnut.2022.1026450 (PMC9806224; doi:10.3389/fnut.2022.1026450)
Supplement: Supplementary file 1 [file Data_Sheet_1.docx]

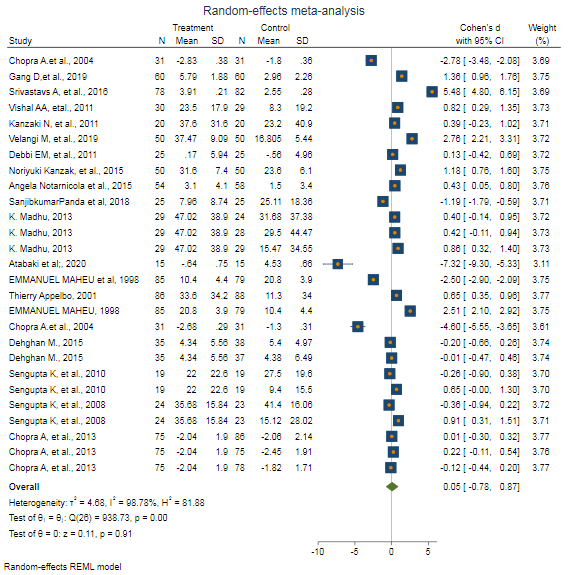


Supplementary figure 1: Forest plot for the association between antioxidants and interested outcomes of OA; based on the visual analogue scale (VAS).


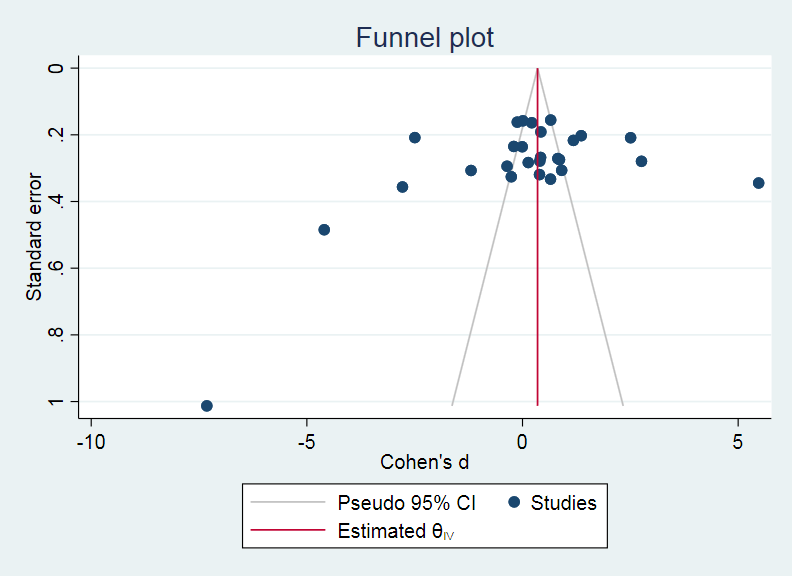


Supplementary figure 2: Funnel plot detailing publication bias in the studies reporting the association between antioxidants and interested outcomes of OA; based on the visual analogue scale (VAS)


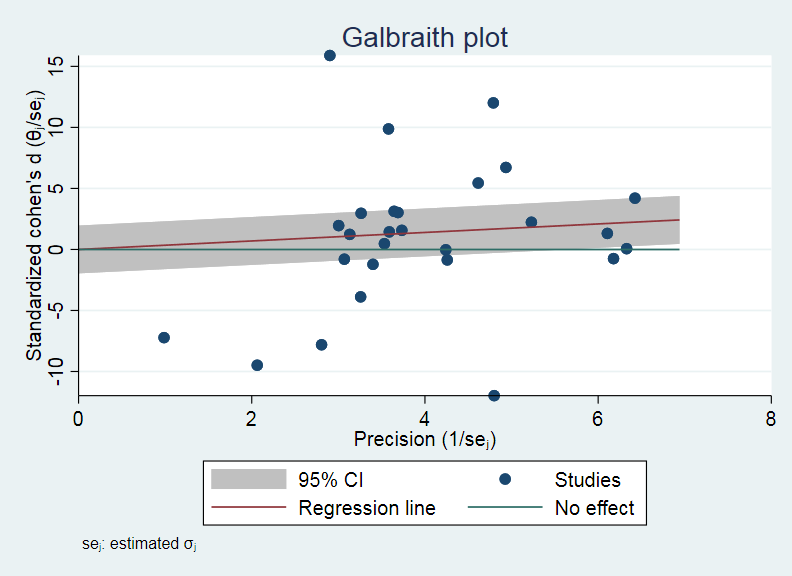


Supplementary figure 3: Galbraith plot detailing heterogeneity in the studies reporting the association between antioxidants and interested outcomes of OA; based on the visual analogue scale (VAS)


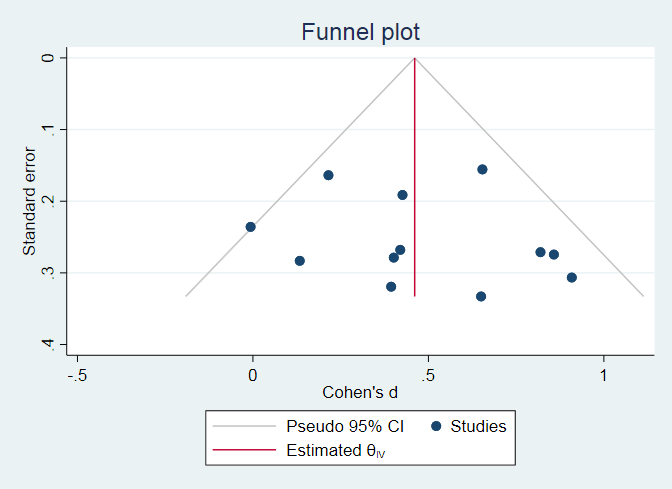


Supplementary figure 4: Funnel plot detailing publication bias in the studies reporting the association between antioxidants and interested outcomes of OA; based on the visual analogue scale (VAS)


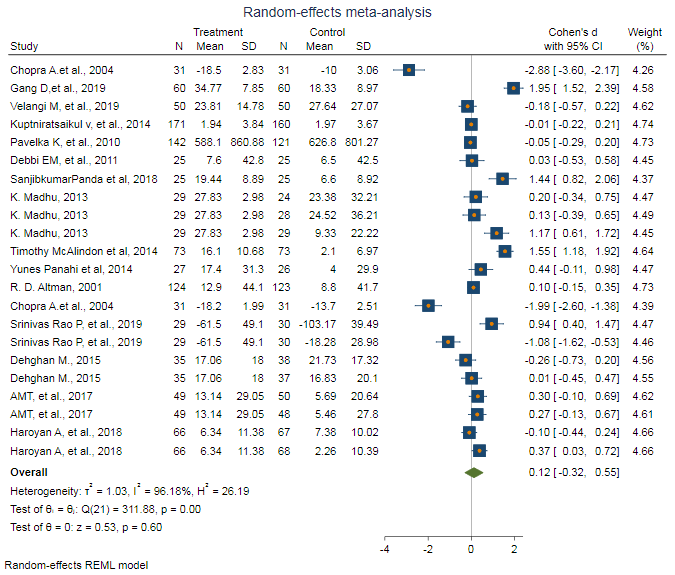


Supplementary figure 5: Forest plot for the association between antioxidants and interested outcomes of OA; based on the Western Ontario and McMaster Osteoarthritis Index (WOMAC) combined pain score.


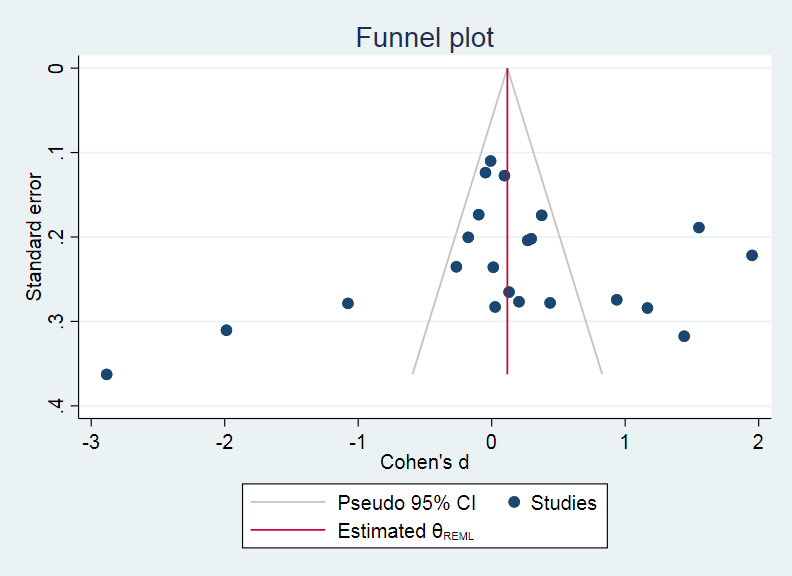


Supplementary figure 6: Funnel plot detailing publication bias in the studies reporting the association between antioxidants and interested outcomes of OA; based on the Western Ontario and McMaster Osteoarthritis Index (WOMAC) combined pain score


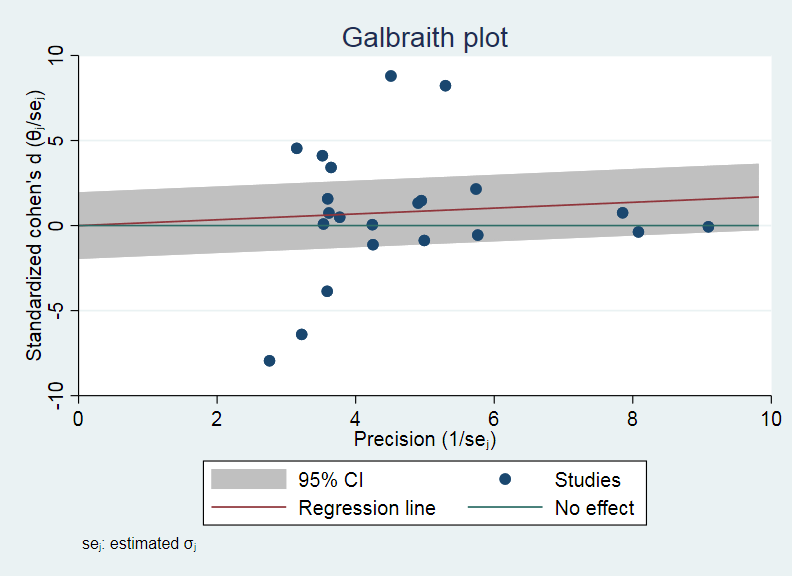


Supplementary figure 7: Galbraith plot detailing heterogeneity in the studies reporting the association between antioxidants and interested outcomes of OA; based on the Western Ontario and McMaster Osteoarthritis Index (WOMAC) combined pain score


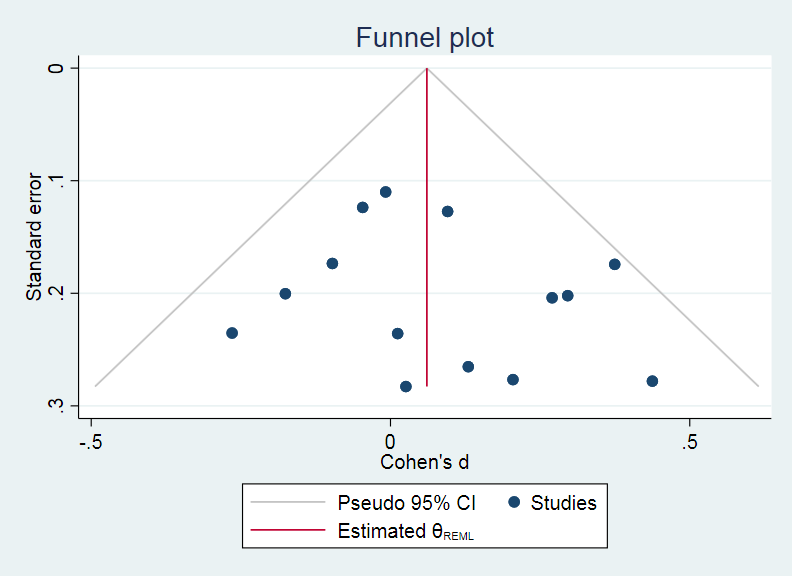


Supplementary figure 8: Funnel plot detailing publication bias in the studies reporting the association between antioxidants and interested outcomes of OA; based on the Western Ontario and McMaster Osteoarthritis Index (WOMAC) combined pain score


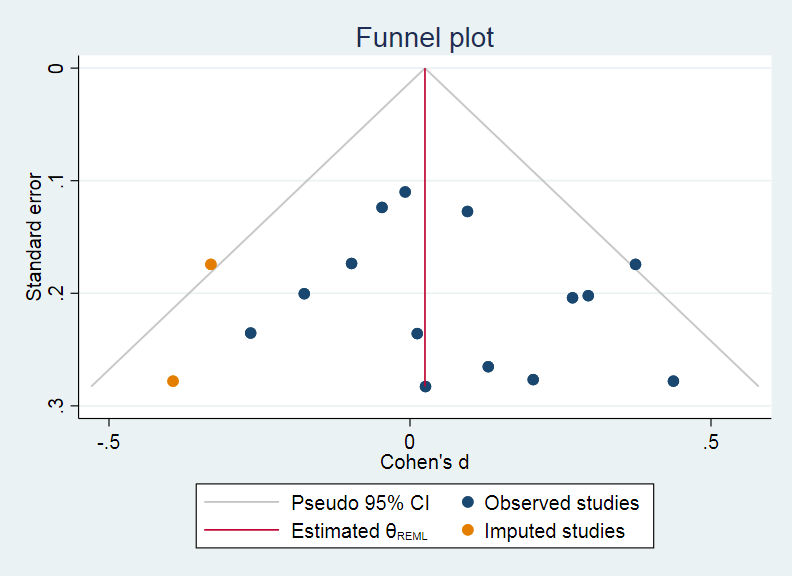


Supplementary figure 9: Funnel plot detailing publication bias in the studies reporting the association between antioxidants and interested outcomes of OA; based on the Western Ontario and McMaster Osteoarthritis Index (WOMAC) combined pain score.


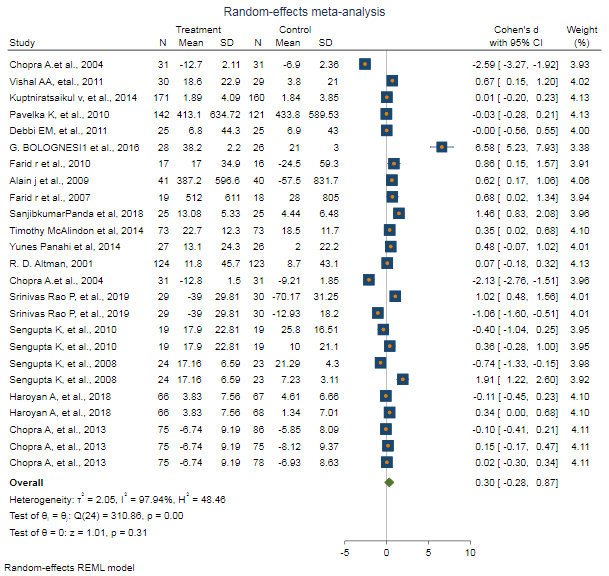


Supplementary figure 10: Forest plot for the association between antioxidants and interested outcomes of OA; based on the Western Ontario and McMaster Osteoarthritis Index (WOMAC) difficulty pain score.


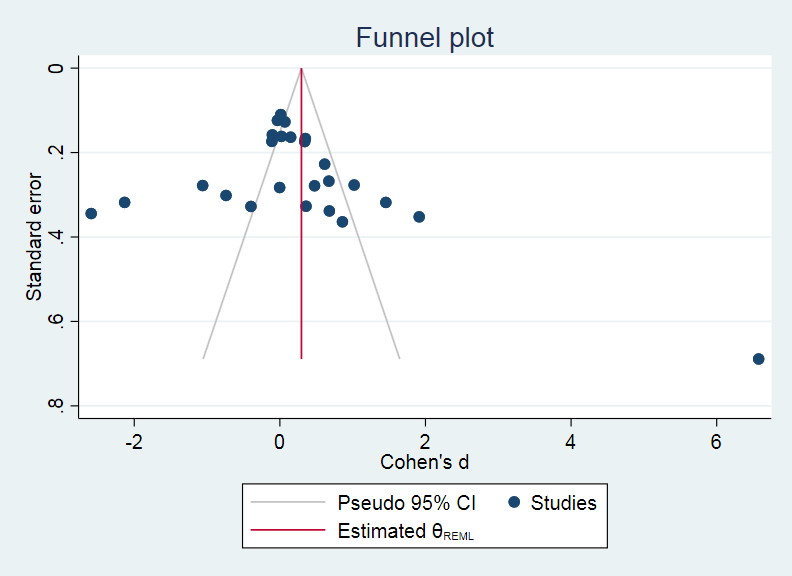


Supplementary figure 11: Funnel plot detailing publication bias in the studies reporting the association between antioxidants and interested outcomes of OA; based on the Western Ontario and McMaster Osteoarthritis Index (WOMAC) difficulty pain score.


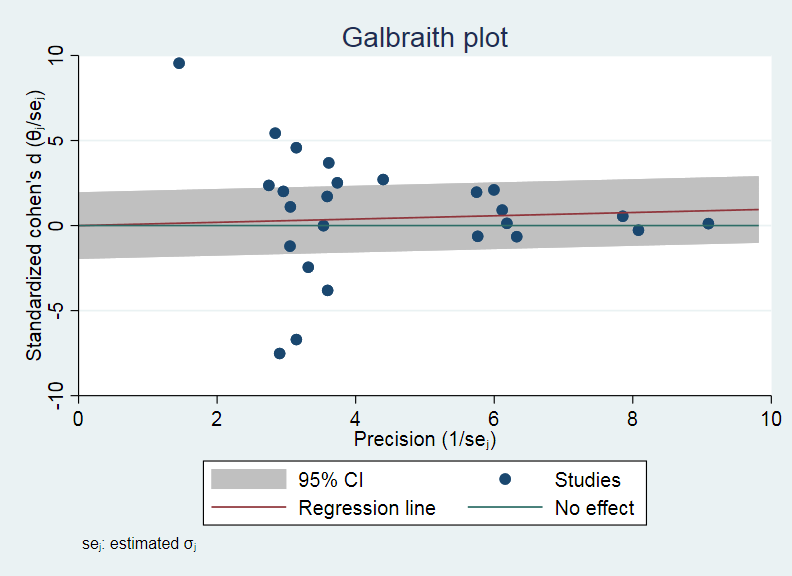


Supplementary figure 12: Galbraith plot detailing heterogeneity in the studies reporting the association between antioxidants and interested outcomes of OA; based on the Western Ontario and McMaster Osteoarthritis Index (WOMAC) difficulty pain score Osteoarthritis Index (WOMAC) difficulty pain score.


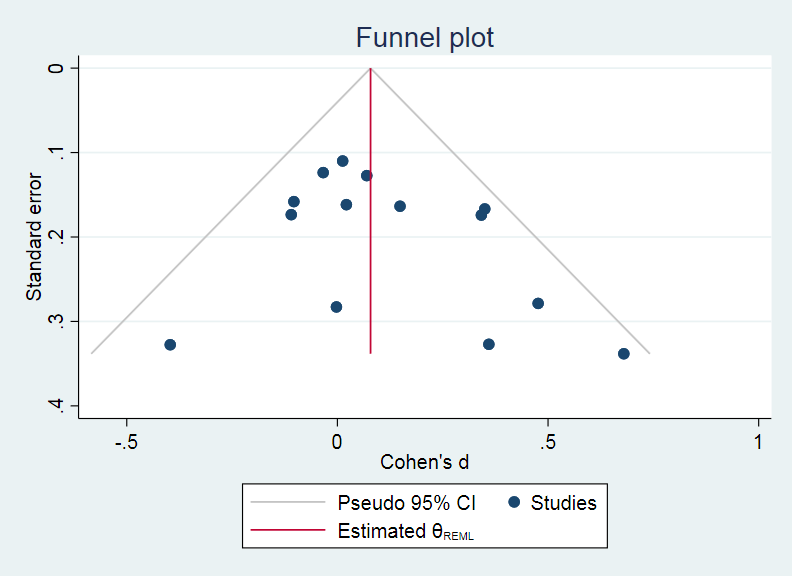


Supplementary figure 13: Funnel plot detailing publication bias in the studies reporting the association between antioxidants and interested outcomes of OA; based on the Western Ontario and McMaster Osteoarthritis Index (WOMAC) difficulty pain score.


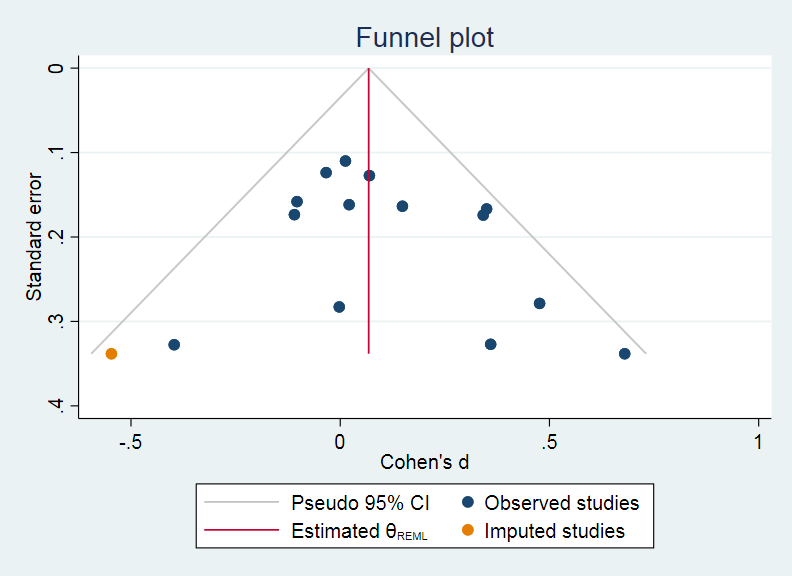


Supplementary figure 14: Funnel plot detailing publication bias in the studies reporting the association between antioxidants and interested outcomes of OA; based on the Western Ontario and McMaster Osteoarthritis Index (WOMAC) difficulty pain score.

.


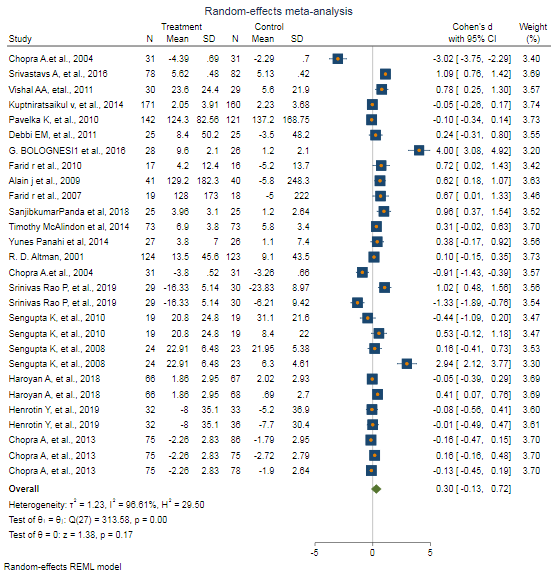


Supplementary figure 15: Forest plot for the association between antioxidants and interested outcomes of OA; based on the Western Ontario and McMaster Osteoarthritis Index (WOMAC) pain score.


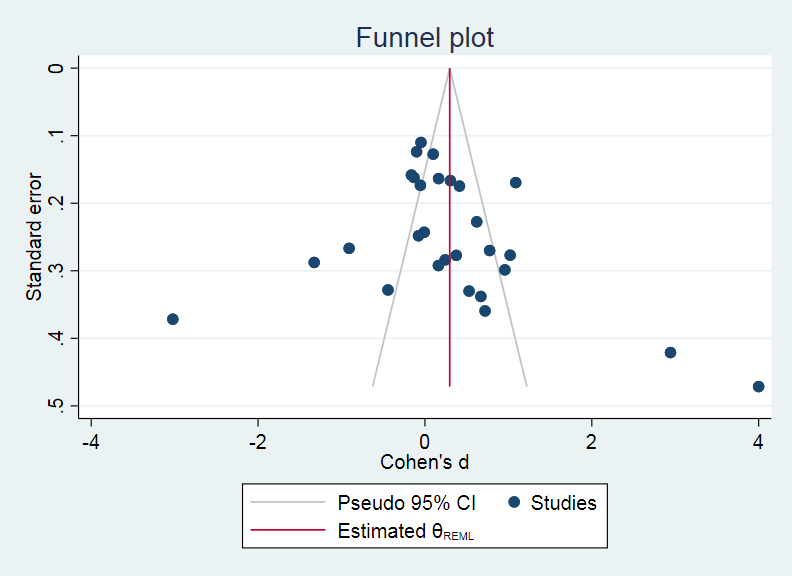


Supplementary figure 16: Funnel plot detailing publication bias in the studies reporting the association between antioxidants and interested outcomes of OA; based on the Western Ontario and McMaster Osteoarthritis Index (WOMAC) pain score.


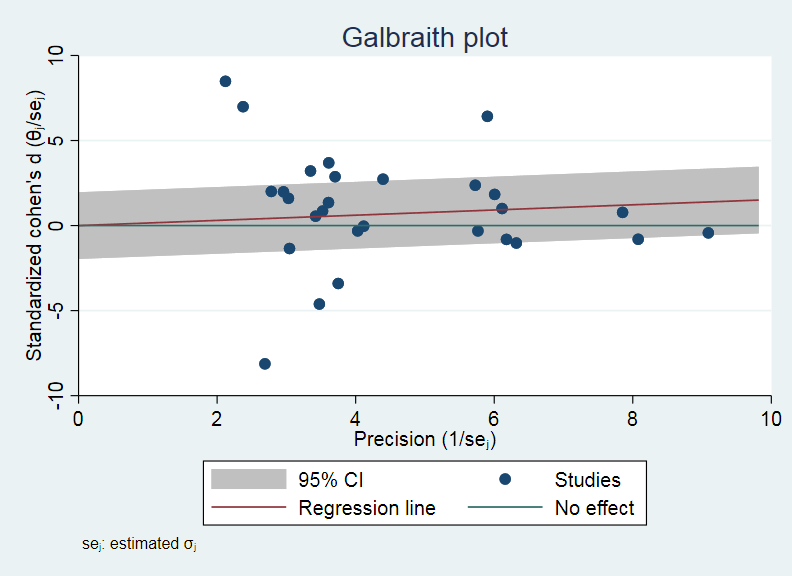


Supplementary figure 17: Galbraith plot detailing heterogeneity in the studies reporting the association between antioxidants and interested outcomes of OA; based on the Western Ontario and McMaster Osteoarthritis Index (WOMAC) pain score.


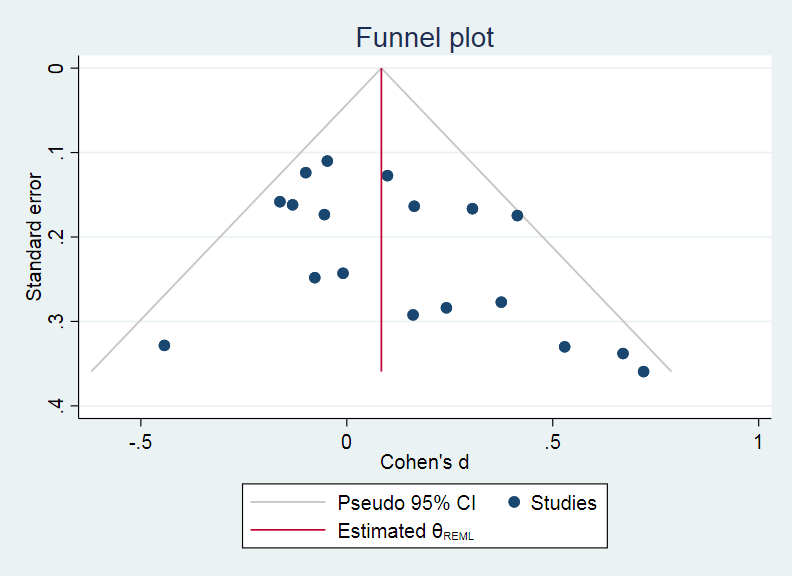


Supplementary figure 18: Funnel plot detailing publication bias in the studies reporting the association between antioxidants and interested outcomes of OA; based on the Western Ontario and McMaster Osteoarthritis Index (WOMAC) pain score.


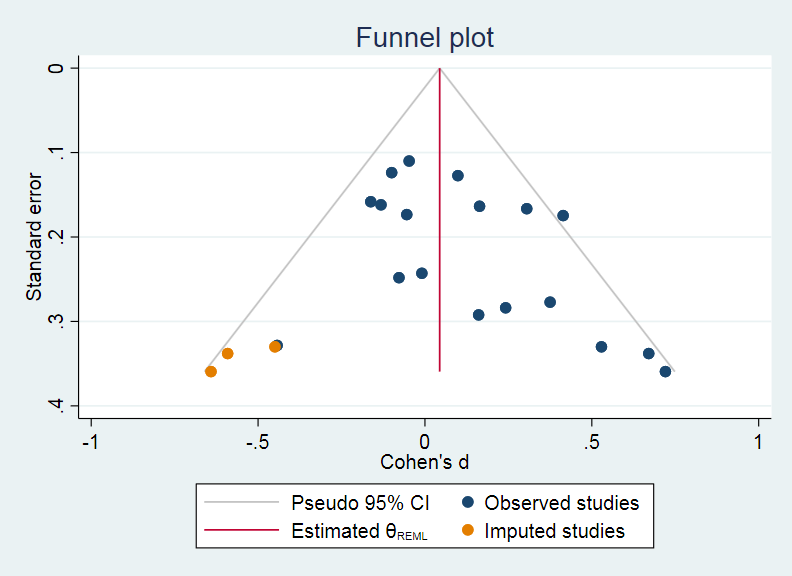


Supplementary figure 19: Funnel plot detailing publication bias in the studies reporting the association between antioxidants and interested outcomes of OA; based on the Western Ontario and McMaster Osteoarthritis Index (WOMAC) pain score.


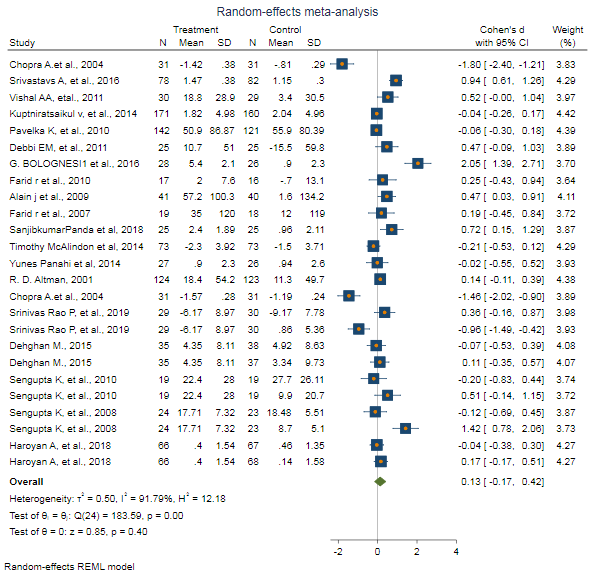


Supplementary figure 20: Forest plot for the association between antioxidants and interested outcomes of OA; based on the Western Ontario and McMaster Osteoarthritis Index (WOMAC) Stiffness score.


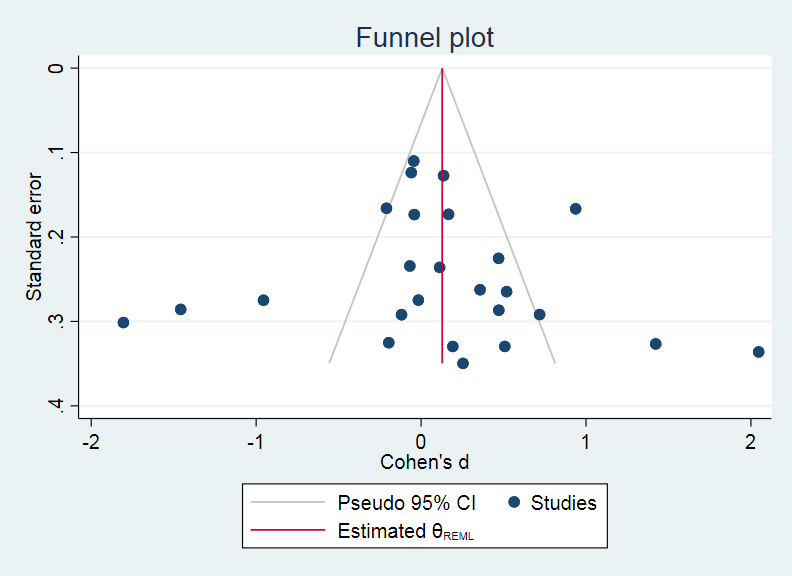


Supplementary figure 21: Funnel plot detailing publication bias in the studies reporting the association between antioxidants and interested outcomes of OA; based on the based on the Western Ontario and McMaster Osteoarthritis Index (WOMAC) Stiffness score.


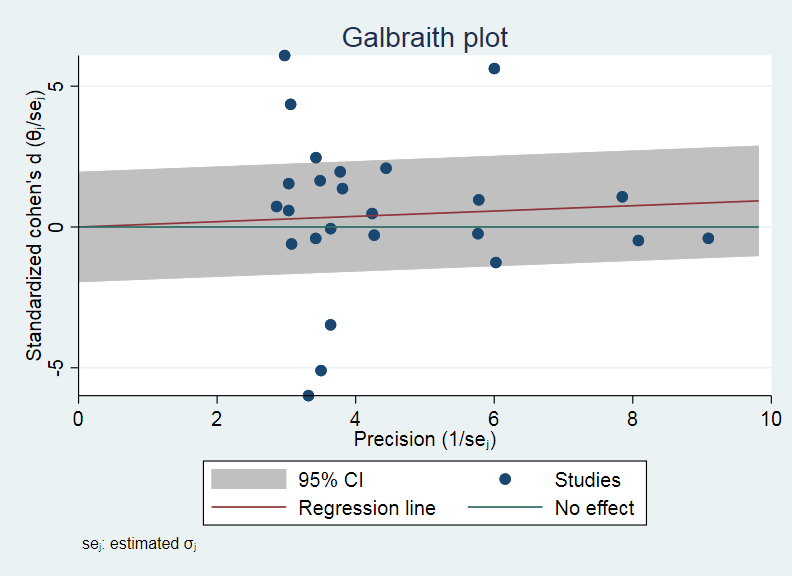


Supplementary figure 22: Galbraith plot detailing heterogeneity in the studies reporting the association between antioxidants and interested outcomes of OA; based on the Western Ontario and McMaster Osteoarthritis Index (WOMAC) Stiffness score.


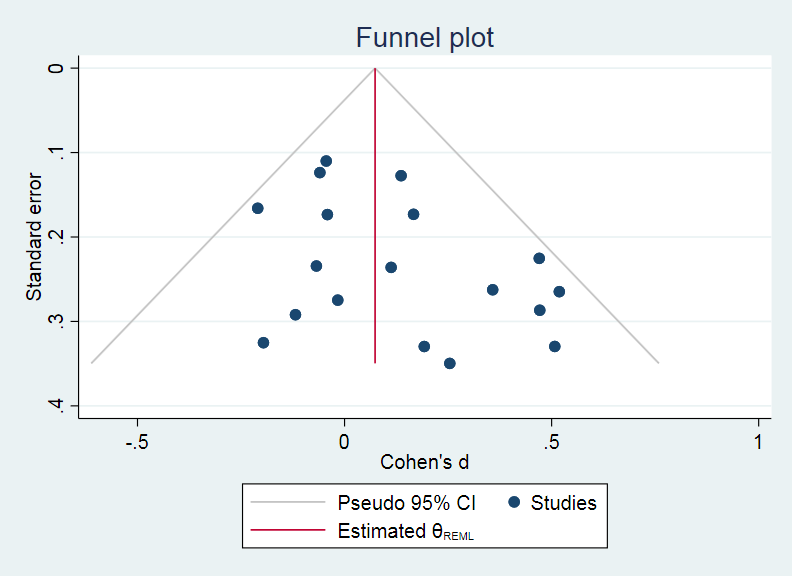


Supplementary figure 23: Funnel plot detailing publication bias in the studies reporting the association between antioxidants and interested outcomes of OA; based on the Western Ontario and McMaster Osteoarthritis Index (WOMAC) Stiffness score.


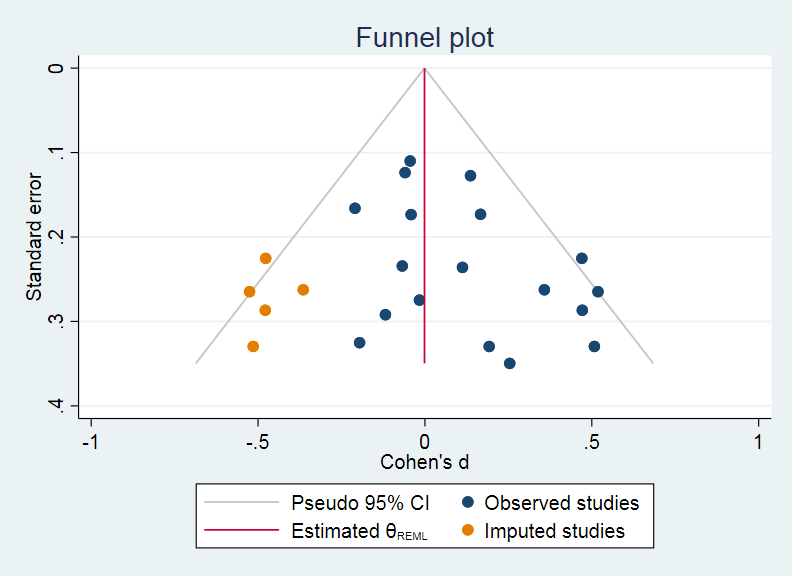


Supplementary figure 24: Funnel plot detailing publication bias in the studies reporting the association between antioxidants and interested outcomes of OA; based on the Western Ontario and McMaster Osteoarthritis Index (WOMAC) Stiffness score.
