# Supplementary material for: The effects of antioxidants on knee osteoarthritis: A systematic review and meta-analysis
Source: Front Nutr. 2022 Dec 19;9:1026450. doi: 10.3389/fnut.2022.1026450 (PMC9806224; doi:10.3389/fnut.2022.1026450)
Supplement: Supplementary file 2 [file Table_1.docx]

| **Supplementary Table 1. Search strategy** |
| --- |
| **PubMed** |
| [((("Osteoarthritis, Knee"[Mesh]) OR "Osteoarthritis"[Mesh]) OR (((((osteoarthritis[Title/Abstract]) OR (Arthritis[Title/Abstract])) OR (Arthritides[Title/Abstract])) OR ("Knee Joint"[Title/Abstract])) OR ("Knee OA"[Title/Abstract]))) AND (((((((((((((("antioxidants"[All Fields] OR "antioxidants"[MeSH Terms] OR antioxidants[Text Word]) OR ("oxidative stress"[MeSH Terms] OR stress oxidative[Text Word])) OR (vitamin C[Title/Abstract])) OR (ascorbate[Title/Abstract])) OR ("vitamin E"[Title/Abstract])) OR (glutathione[Title/Abstract])) OR (Turmeric[Title/Abstract])) OR (Curcuma[Title/Abstract])) OR (Piperine[Title/Abstract])) OR (Avocado[Title/Abstract])) OR (Boswellia[Title/Abstract])) OR (Superoxide[Title/Abstract])) OR (Peroxide[Title/Abstract])) OR (Peroxynitrite[Title/Abstract]))](https://pubmed.ncbi.nlm.nih.gov/?term=%28%28%28%22Osteoarthritis%2C+Knee%22%5BMesh%5D%29+OR+%22Osteoarthritis%22%5BMesh%5D%29+OR+%28%28%28%28%28osteoarthritis%5BTitle%2FAbstract%5D%29+OR+%28Arthritis%5BTitle%2FAbstract%5D%29%29+OR+%28Arthritides%5BTitle%2FAbstract%5D%29%29+OR+%28%22Knee+Joint%22%5BTitle%2FAbstract%5D%29%29+OR+%28%22Knee+OA%22%5BTitle%2FAbstract%5D%29%29%29+AND+%28%28%28%28%28%28%28%28%28%28%28%28%28%28%22antioxidants%22%5BAll+Fields%5D+OR+%22antioxidants%22%5BMeSH+Terms%5D+OR+antioxidants%5BText+Word%5D%29+OR+%28%22oxidative+stress%22%5BMeSH+Terms%5D+OR+stress+oxidative%5BText+Word%5D%29%29+OR+%28vitamin+C%5BTitle%2FAbstract%5D%29%29+OR+%28ascorbate%5BTitle%2FAbstract%5D%29%29+OR+%28%22vitamin+E%22%5BTitle%2FAbstract%5D%29%29+OR+%28glutathione%5BTitle%2FAbstract%5D%29%29+OR+%28Turmeric%5BTitle%2FAbstract%5D%29%29+OR+%28Curcuma%5BTitle%2FAbstract%5D%29%29+OR+%28Piperine%5BTitle%2FAbstract%5D%29%29+OR+%28Avocado%5BTitle%2FAbstract%5D%29%29+OR+%28Boswellia%5BTitle%2FAbstract%5D%29%29+OR+%28Superoxide%5BTitle%2FAbstract%5D%29%29+OR+%28Peroxide%5BTitle%2FAbstract%5D%29%29+OR+%28Peroxynitrite%5BTitle%2FAbstract%5D%29%29&filter=hum_ani.humans) |
| **Scopus** |
| ( ( TITLE-ABS-KEY ( osteoarthrit* )  OR  TITLE-ABS-KEY ( arthritis )  OR  TITLE-ABS-KEY ( "Knee Joint" ) ) )  AND  ( ( TITLE-ABS-KEY ( antioxidant* )  OR  TITLE-ABS-KEY ( "oxidative stress" )  OR  TITLE-ABS-KEY ( "stress oxidative" )  OR  TITLE-ABS-KEY ( "vitamin C" )  OR  TITLE-ABS-KEY ( ascorbate )  OR  TITLE-ABS-KEY ( "vitamin E" )  OR  TITLE-ABS-KEY ( "glutathione" )  OR  TITLE-ABS-KEY ( curcuma )  OR  TITLE-ABS-KEY ( piperine )  OR  TITLE-ABS-KEY ( avocado )  OR  TITLE-ABS-KEY ( boswellia )  OR  TITLE-ABS-KEY ( superoxide )  OR  TITLE-ABS-KEY ( peroxide )  OR  TITLE-ABS-KEY ( peroxynitrite ) ) )  AND  ( LIMIT-TO ( PUBSTAGE ,  "final" ) )  AND  ( LIMIT-TO ( DOCTYPE ,  "ar" ) )  AND  ( LIMIT-TO ( SUBJAREA ,  "MEDI" ) )  AND  ( LIMIT-TO ( SRCTYPE ,  "j" ) ) |
| **ISI/WOS** |
| TOPIC: ("osteoarthrit*") OR TOPIC: ("arthritis ") OR TOPIC: ("Knee Joint ")  Indexes=SCI-EXPANDED, SSCI, CPCI-S, CPCI-SSH, ESCI Timespan=All years  TOPIC: (antioxidant*) OR TOPIC: (oxidative stress) OR TOPIC: ("vitamin C ") OR TOPIC: (ascorbate) OR TOPIC: ("vitamin E") OR TOPIC: ("glutathione ") OR TOPIC: ("curcuma") OR TOPIC: ("piperine ") OR TOPIC: ("avocado ") OR TOPIC: ("boswellia ") OR TOPIC: ("superoxide ") OR TOPIC: ("peroxide ") OR TOPIC: ("peroxynitrite ")  Indexes=SCI-EXPANDED, SSCI, CPCI-S, CPCI-SSH, ESCI Timespan=All years  #2 AND #1  Indexes=SCI-EXPANDED, SSCI, CPCI-S, CPCI-SSH, ESCI Timespan=All years |

Supplementary table 1. Search strategy
