# Supplementary material for: The effects of antioxidants on knee osteoarthritis: A systematic review and meta-analysis
Source: Front Nutr. 2022 Dec 19;9:1026450. doi: 10.3389/fnut.2022.1026450 (PMC9806224; doi:10.3389/fnut.2022.1026450)
Supplement: Supplementary file 3 [file Table_2.docx]

**Supplementary table 2**. Cochrane collaborator tools for risk of bias assessment of included papers

| **NO** | **Citation** | **Random sequence generation** | **Allocation concealment** | **Blinding of participants and personnel** | **Blinding of outcome data** | **Incomplete outcome data** | **Selective reporting** | **Other bias** |
| --- | --- | --- | --- | --- | --- | --- | --- | --- |
| 1 | Gang D, et al; 2019(36) | + | + | - | - | + | + | + |
| 2 | Srivastavs A, et al; 2016(37) | + | + | - | - | + | + | + |
| 3 | Vishal AA, et al 2011(38) | + | + | ˟ | - | + | ˟ | + |
| 4 | Kanzaki N, et al; 2011(39) | + | ˟ | + | - | + | + | + |
| 5 | Velangi M, et al; 2019(40) | ˟ | + | + | - | + | ˟ | + |
| 6 | Kuptniratsaikul v, et al; 2014(41) | + | + | - | - | + | ˟ | + |
| 7 | Pavelka K, et al; 2010(27) | + | + | - | - | + | + | + |
| 8 | Debbi EM, et al; 2011(42) | + | ˟ | ˟ | - | + | ˟ | + |
| 9 | Kanzak, N,et al; 2015(43) | + | + | - | - | + | + | + |
| 10 | Notarnicola A,et al; 2015(44) | + | ˟ | + | - | + | + | + |
| 11 | Bolognesi G ,et al; 2016.(35) | + | ˟ | + | - | + | + | + |
| 12 | Farid r, et al; 2010(45) | + | + | - | - | + | + | + |
| 13 | Jacquet A, et al;2009(13) | ˟ | + | - | - | + | + | + |
| 14 | Farid R ,et al; 2007(46) | + | ˟ | - | - | + | + | + |
| 15 | Panda S, et al; 2018(47) | + | + | + | - | + | + | + |
| 16 | Madhu K,2013;(33) | + | + | + | - | + | + | + |
| 17 | Madhu K,2013;(33) | + | + | + | - | + | + | + |
| 18 | Madhu K, et al; 2013(33) | + | + | + | - | + | + | + |
| 19 | Atabaki et al, 2020;(8) | + | ˟ | - | - | + | ˟ | ˟ |
| 20 | Maheu E, et al,1998;(48) | + | + | + | - | + | + | + |
| 21 | Timothy McAlindon et al,2014;(34) | + | + | + | - | + | + | + |
| 22 | Panahi Y,et al; 2014(49) | + | + | + | - | + | + | + |
| 23 | Altman R. D,et al;2001(50) | + | + | - | - | + | + | + |
| 24 | Appelbo T, et al; 2001(51) | + | + | - | - | + | + | + |
| 25 | Appelbo T, et al; 2001(51) | + | + | - | - | + | + | + |
| 26 | Chopra A,et al; 2004(31) | + | + | + | - | + | ˟ | ˟ |
| 27 | Srinivas Rao P, et al;2019(28) | + | + | + | - | + | + | + |
| 28 | Srinivas Rao P, et al; 2019(28) | + | + | + | - | + | + | + |
| 29 | Dehghan M, et al; 2015(52) | + | + | - | - | + | ˟ | ˟ |
| 30 | Dehghan M, et al; 2015(52) | + | + | - | - | + | ˟ | ˟ |
| 31 | Sengupta K, et al; 2010(29) | + | ˟ | ˟ | - | + | + | + |
| 32 | Sengupta K, et al; 2010(29) | + | ˟ | ˟ | - | + | + | + |
| 33 | Lubis AMT, et al; 2017(53) | ˟ | + | - | - | + | + | + |
| 34 | Lubis AMT, et al; 2017(53) | ˟ | + | - | - | + | + | + |
| 35 | Sengupta K, et al; 2008(7) | + | + | - | - | + | + | + |
| 36 | Sengupta K, et al; 2008(7) | + | + | - | - | + | + | + |
| 37 | Haroyan A, et al; 2018(54) | + | + | ˟ | - | + | + | ˟ |
| 38 | Haroyan A, et al; 2018(54) | + | + | ˟ | - | + | + | ˟ |
| 39 | Henrotin Y, et al. 2019;(32) | + | + | ˟ | - | + | + | + |
| 40 | Henrotin Y, et al. 2019;(32) | + | + | ˟ | - | + | + | + |
| 41 | Chopra A, et al, 2013;(55) | + | + | - | - | + | + | + |
| 42 | Chopra A, et al, 2013;(55) | + | + | - | - | + | + | + |
| 43 | Chopra A, et al, 2013;(55) | + | + | - | - | + | + | + |

+ Low risk of bias

˟ Unclear risk of bias

- High risk of bias
